# Structure-based insights into evolution of rhodopsins

**DOI:** 10.1038/s42003-021-02326-4

**Published:** 2021-06-30

**Authors:** Dmitrii Zabelskii, Natalia Dmitrieva, Oleksandr Volkov, Vitaly Shevchenko, Kirill Kovalev, Taras Balandin, Dmytro Soloviov, Roman Astashkin, Egor Zinovev, Alexey Alekseev, Ekaterina Round, Vitaly Polovinkin, Igor Chizhov, Andrey Rogachev, Ivan Okhrimenko, Valentin Borshchevskiy, Vladimir Chupin, Georg Büldt, Natalia Yutin, Ernst Bamberg, Eugene Koonin, Valentin Gordeliy

**Affiliations:** 1 Institute of Biological Information Processing (IBI-7: Structural Biochemistry), ForschungszentrumJülich, Jülich, Germany; 2 JuStruct: Jülich Center for Structural Biology, Forschungszentrum Jülich, Jülich, Germany; 3 Research Center for Molecular Mechanisms of Aging and Age-related Diseases, Moscow Institute of Physics and Technology, Dolgoprudny, Russia; 4 Institut für Biologische Informationsprozesse – Molekular- und Zellphysiologie (IBI-1),Forschungszentrum Jülich, Jülich, Germany; 5 Institute of Crystallography, University of Aachen (RWTH), Aachen, Germany; 6 Institut de Biologie Structurale (IBS), Université Grenoble Alpes-CEA-CNRS, Grenoble, France; 7 European Molecular Biology Laboratory, Hamburg unit c/o DESY, Hamburg, Germany; 8 Joint Institute for Nuclear Research, Dubna, Russia; 9 Institute for Safety Problems of Nuclear Power Plants, NAS of Ukraine, Kyiv, Ukraine; 10 European Molecular Biology Laboratory, Hamburg Unit, Notkestrasse 25a, Hamburg, Germany; 11 ELI Beamlines, Institute of Physics, Czech Academy of Sciences, Prague, Czech Republic; 12 Institute for Biophysical Chemistry, Hannover Medical School, Hannover, Germany; 13 National Center for Biotechnology Information, National Library of Medicine, National Institutesof Health, Bethesda, MD United States; 14 Max Planck Institute of Biophysics, Frankfurt am Main, Germany

**Keywords:** Ion transport, Molecular evolution

## Abstract

Rhodopsins, most of which are proton pumps generating transmembrane electrochemical proton gradients, span all three domains of life, are abundant in the biosphere, and could play a crucial role in the early evolution of life on earth. Whereas archaeal and bacterial proton pumps are among the best structurally characterized proteins, rhodopsins from unicellular eukaryotes have not been well characterized. To fill this gap in the current understanding of the proton pumps and to gain insight into the evolution of rhodopsins using a structure-based approach, we performed a structural and functional analysis of the light-driven proton pump LR (Mac) from the pathogenic fungus *Leptosphaeria maculans*. The first high-resolution structure of fungi rhodopsin and its functional properties reveal the striking similarity of its membrane part to archaeal but not to bacterial rhodopsins. We show that an unusually long N-terminal region stabilizes the protein through direct interaction with its extracellular loop (ECL2). We compare to our knowledge all available structures and sequences of outward light-driven proton pumps and show that eukaryotic and archaeal proton pumps, most likely, share a common ancestor.

## Abstract (web summary)

Zabelskii et al. present a structural and functional analysis of the lightdriven proton pump LR (Mac) from the fungus Leptosphaeria maculans. Their findings indicate that the archaeal ancestry of eukaryotic type 1 rhodopsins, and that the archaeal host of the proto-mitochondrial endosymbiont was capable of light-driven proton pumping.

## Introduction

Microbial (type 1) rhodopsins are the most abundant family of light-harvesting proteins. Type 1 rhodopsins are heptahelical transmembrane (7TM) proteins that covalently bind the retinal chromophore and use the energy of light to perform different biological functions, such as ion pumping^1–6^, ion channeling^7–9^, sensoring^10–12^, and kinase activity^13^. The explosion of research on microbial rhodopsins, in large part, owes to the key role of these proteins in optogenetics, a methodology that caused a revolution in neuroscience^14,15^. Recently, discoveries of genomics and metagenomics show that rhodopsins are highly abundant, perform extremely diverse functions, and are present in all kingdoms of life as well as many large viruses^16,17^. Rhodopsins are considered to be the most abundant light-harvesting proteins on earth and the major light capturers in the oceans^18^. Given the ubiquity of rhodopsins and their crucial ecological role, there is little doubt that these proteins played a crucial role in the evolution of life on earth. Recently, rhodopsins have been identified in Asgard archaea^19^, the archaeal superphylum that includes the likely ancestors of eukaryotes^20,21^. Furthermore, a recent analysis of microbial rhodopsins has led to the hypothesis that retinal-based phototrophy emerged early in the evolution of life on Earth, predating the rise and profoundly impacting the evolution of photosynthesis^22^. Thus, the study of the functions and evolution of rhodopsins could yield valuable information on the origin and early evolution of life.

Among the factors critical in the evolution of early life was the ability to convert the energy of sunlight into a transmembrane proton gradient that provides for chemiosmotic coupling^23^. Light-driven proton pumps generating transmembrane gradients are the most abundant among the rhodopsins^24^. The structure and function of these proteins are exceptionally well studied, providing a rare opportunity to use a structure-based approach to explore evolutionary relationships among proteins.

Whereas structures of archaeal and bacterial proton pumps have been thoroughly characterized, this is not the case for rhodopsins of unicellular eukaryotes. To fill this gap and enable a structure-based analysis of the evolutionary relationships among the rhodopsins, we determined a high-resolution structure and performed an in-depth functional study of a light-driven proton pump LR (Mac) from the fungus *Leptosphaeria maculans*. *Leptosphaeria maculans* is a major pathogen of *Brassica napus*, an agricultural plant that is used as a feed source for livestock and the production of rapeseed oil. A dramatic epidemic of *L. maculans* occurred in Wisconsin on cabbage. The fungus destroys around 5–20% of canola yields in France^25^. The disease is also harmful in England^26^. Rapeseed oil is the preferred European oil source for biofuel due to its high yield. *B. napus* produces more oil per land area than other sources like soybeans. Thus, apparent from the fundamental importance of a thorough characterization of rhodopsin from a unicellular eukaryote, the study of LR (Mac) also could help understand the role of the rhodopsin in the fungus pathogenicity.

To date, high-resolution structures of multiple proton-pumping archaeal and bacterial rhodopsins have been solved^27–30^. For instance, more than 100 structures of the most-studied microbial rhodopsin, bacteriorhodopsin from archaeon *Halobacterium salinarum* (*Hs*BR), and its mutants, were deposited to the Protein Data Bank (PDB) since 1997^31^. By contrast, structural characterization of eukaryotic type-1 rhodopsins lags far behind. Two high-resolution structures of an H^+^ pumping rhodopsin from unicellular eukaryotes are currently available, Acetabularia rhodopsin (AR) from the marine alga *Acetabularia acetabulum*^32,33^ and Coccomyxa rhodopsin from *Coccomyxa subellipsoidea*^34^. The AR has been deemed to be closely similar to archaeal *Hs*BR although this protein has a 200 ms long photocycle, compared to the 20 ms photocycle in *Hs*BR^35^. High-resolution structures of eukaryotic proton-pumping rhodopsins from fungi would be crucial for understanding the evolution of this expansive protein superfamily.

The study of eukaryotic membrane proteins is generally hampered by the complications with their expression and crystallization. We used our recently developed techniques of the expression in LEXSY^36^ and crystallized the proton-pumping type 1 rhodopsin LR from the unicellular fungus *Leptosphaeria maculans*. Previously, biophysical features of LR have been reported to resemble those of archaeal pump *Hs*BR^37^ with millisecond-length photocycle and similar active center organization^38^. However, unlike archaeal rhodopsins, LR and other eukaryotic rhodopsins have elongated *N*- and *C*-terminal regions that could be involved in the adaptation of the protein to different lipid environment^39^. Replacement of the LR N-terminal region did not impair the structural integrity and biochemical functionality of the protein, but the direct impact of modifications of the N-terminus has not been reported^38^. Recently, a full-length LR has been shown to enable efficient neural silencing by blue light in mammalian cells^40^.

Here we present the first high-resolution, at 2.2 Å, crystal structure of a light-driven H^+^ pump from fungi and elucidate the functional role of the N-terminal region. We further report the results of a structure-based comparison of LR with light-driven H^+^ pumps from all domains of life. This structural analysis revealed the archaeal ancestry of eukaryotic type 1 rhodopsins. Further structure-based phylogenetic analysis confirmed the archaeal affinity of eukaryotic proton-pumping rhodopsins, suggesting that the archaeal host of the proto-mitochondrial endosymbiont was capable of light-driven proton pumping. Besides that, LR is the second membrane protein after *Cr*ChR2^36^, which was expressed in LEXSY system, further crystallized, and the structure was solved at high-resolution. Therefore, LEXSY expression system might be a reasonable alternative for the expression of eukaryotic membrane proteins in structural studies and, more specifically, eukaryotic microbial rhodopsins.

## Results and discussion

### Overall LR structure and function

We performed a comparative functional characterization of the full-length (residues 1–313) LR and its previously partially characterized N-terminally truncated version (residues 49–313). These proteins did not show any detectable expression in *E. coli*, and therefore, we expressed them in *Leishmania tarentolae* (LEXSY) as previously described for channelrhodopsin 2 (ChR2)^36^ (see “Methods” for details).

First, we describe the structure of the full-length protein, which will be the structural basis for understanding the differences in the properties of rhodopsins and their evolution. LR was crystallized with an *in meso* approach similar to that used previously^5,6^. The structure was solved with crystals grown at pH 7.0 by the molecular replacement method, using the coordinates of *Hs*BR (PDB ID: 1C3W), and refined to 2.2 Å resolution (Table 1). The crystals belong to the P21 space group and comprise two protomers in an asymmetric unit. LR (residues 42–286), all-trans-retinal (ATR), 19 lipid molecules, and 65 water molecules inside the protein are clearly resolved in the electron-density map, whereas the N- and C-terminal domains lack resolution for 41 and 31 residues, respectively (Supplementary Figures 1–2).

**Table 1:** Crystallographic data collection and refinement statistics.

Data collection	LR 1–313
Space group	P 21 21 21
***Cell dimensions***	
*a*, *b*, *c* (Å)	63.54, 70.78, 148.02
*α, β, γ* (0)	90, 90, 90
Wavelength (Å)	0.976
Resolution (Å)	48.21–2.2 (2.26–2.20)
*R*_merge_ (%)	19.4 (139.8)
*I*/σ*I*	5.7 (1.1)
*CC*_*1/2*_ (%)	99.7 (80.4)
Completeness (%)	99.8 (99.6)
Unique reflections	34,622 (2526)
Multiplicity	6.4 (6.2)
***Refinement***	
Resolution (Å)	19.92–2.20
No. reflections	34,340
*R*_work_/*R*_free_ (%)	23.8/28.5
No. atoms	
Protein	3650
Retinal cofactor	40
Water	104
Lipids	419
B-factors (Å²)	
Protein	35
Retinal	27
Water	40
Lipids	44
***R.m.s. deviations***	
Bond lengths (Å)	0.007
Bond angles (0)	0.935

**Fig. 1:**
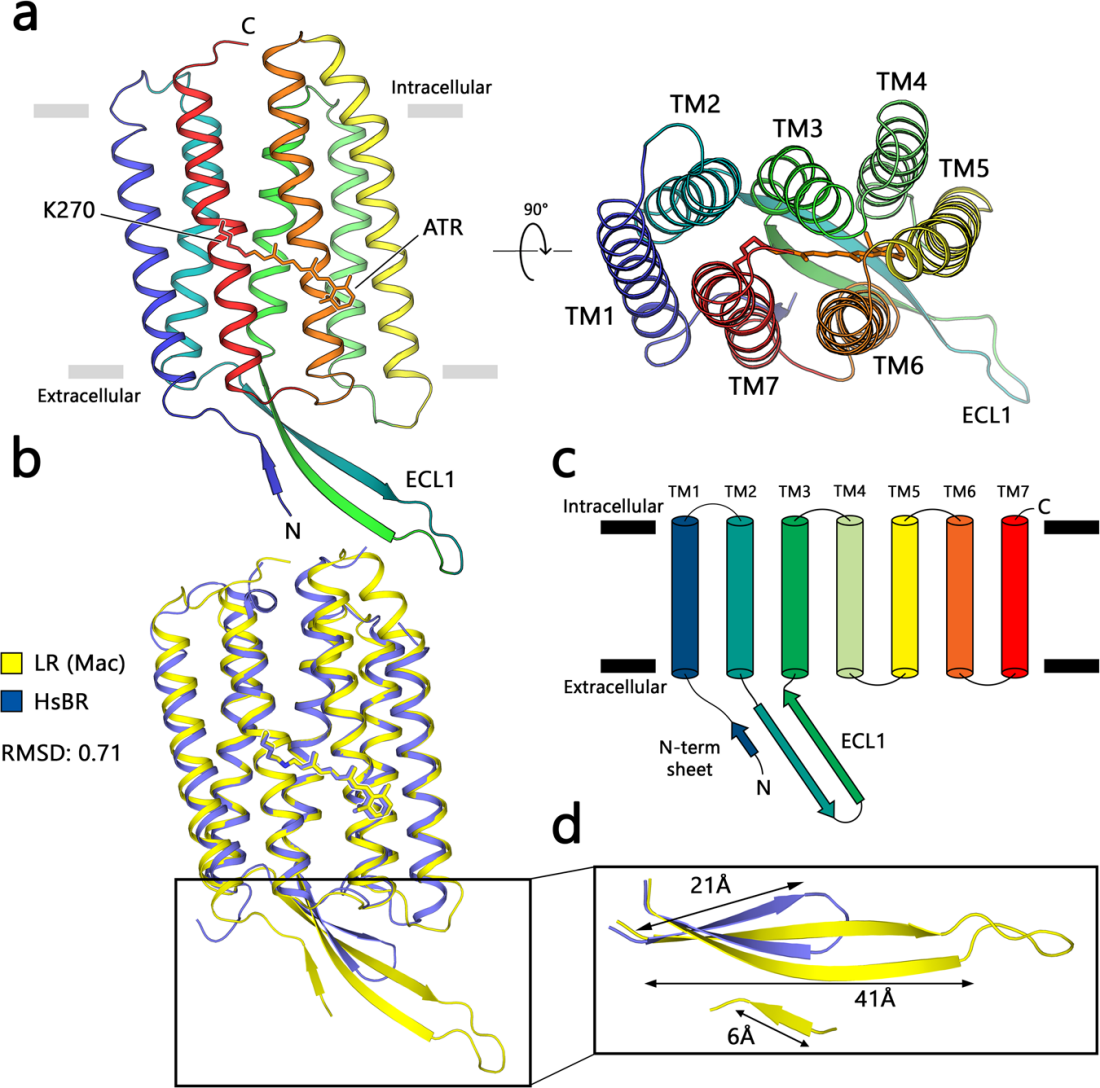
The overall architecture of LR. **a** Crystal structure of LR at pH 7.0 at two different projections. Hydrophobic/hydrophilic interface was calculated with PPM server^83^ and is shown as gray lines, all-trans-retinal (ATR) cofactor is colored orange. **b** Structural superimposing of LR and *Hs*BR (PDB code: 1C3W) structures. **c** Schematic representation of the ribbon diagram. **d** Structural comparison of extracellular loop 1 (ECL1) of LR and *Hs*BR proteins.

The LR bundle consists of seven transmembrane helices-TM1–TM7, connected by three intracellular (ICL 1–3) and three extracellular (ECL 1–3) loops, with retinal chromophore covalently attached to the K270 on the TM7 (Fig. 1). The overall structure of LR aligns well with the archaeal pump *Hs*BR^37^ (PDB ID: 1C3W) and eukaryotic pumps ARII^32^ (PDB ID: 5AWZ) and CsR^34^ (PDB ID: 6GYH), with root mean square deviations (RMSD) of 0.65, 0.72, and 0.76 Å respectively (Supplementary Figure 3). The interior portions of LR and *Hs*BR are virtually identical structurally, and the only major difference is observed at the extracellular sides of these rhodopsins (Fig. 1). Specifically, ECL1 of LR is much longer, consisting of 30 amino acids, whereas the corresponding loop in *Hs*BR contains only 16 residues, which results in a major difference in length, 44 Å in LR vs 21 Å in *Hs*BR. Interestingly, ECL1 of LR is reminiscent of the highly conservative ICL1 beta-sheet domain of heliorhodopsins, which was previously shown to play an important role in dimer formation^41,42^ (Supplementary Figure 4).

### Structural features of LR important for proton transport

**Fig. 2:**
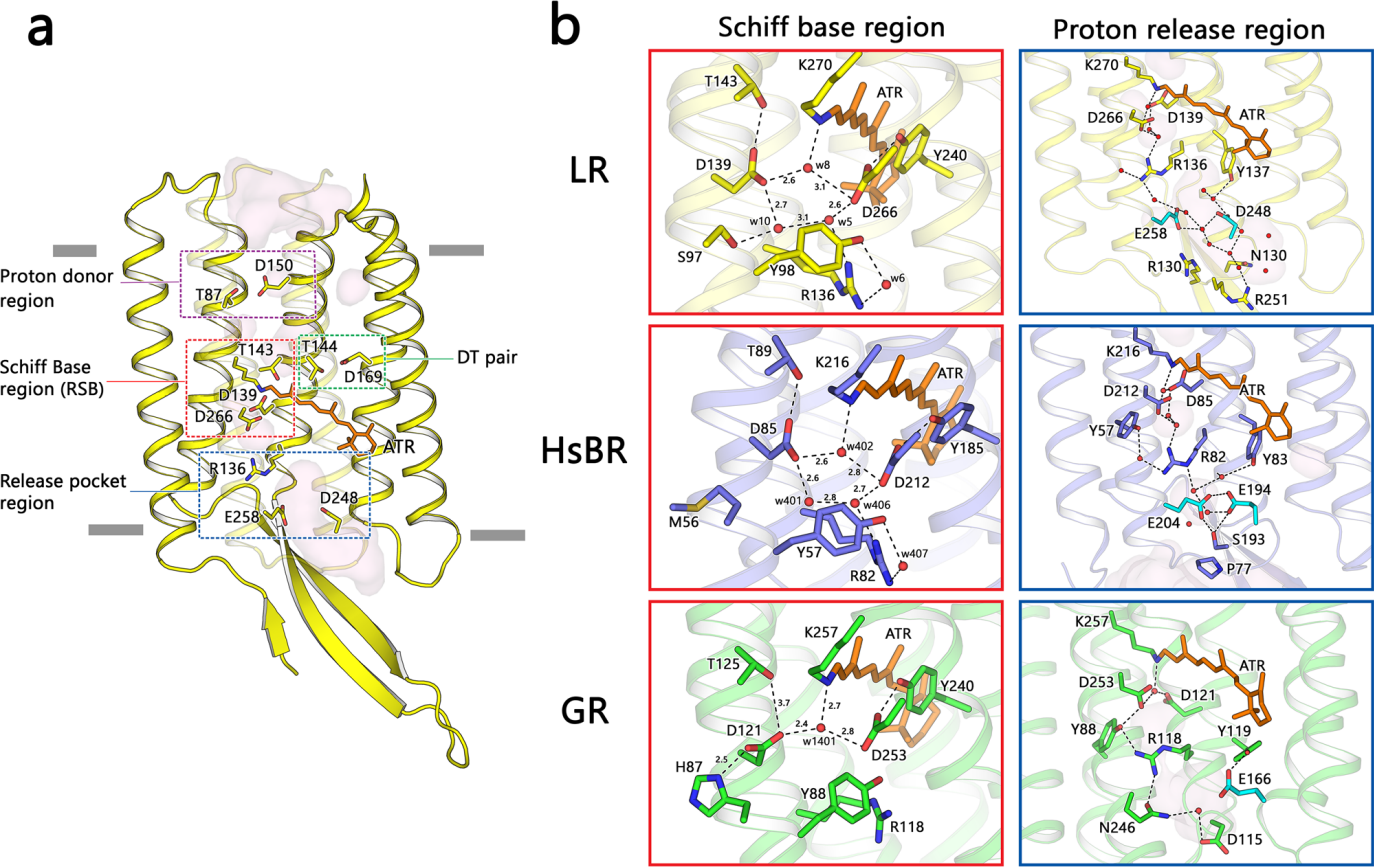
LR proton translocation pathway. **a** Four major regions of LR are involved in the proton transport function of the protein. Functionally important residues are shown with sticks and indicated. **b** Magnified view of retinal Schiff base (RSB) and proton-release pocket regions in LR (this study), archaeal pump *Hs*BR (PDB ID: 1C3W), and bacterial pump GR (PDB ID: 6NWD). The all-trans-retinal is colored orange for all the proteins.

In the LR structure, the retinal chromophore is fixed in an all*-trans* conformation. The protonated retinal Schiff base (RSB) points toward the extracellular side and donates a hydrogen bond to the w8 molecule (Fig. 2a). This arrangement is stabilized by the RSB counterions, D266 and D139, which are counterparts to D212 and D85 in *Hs*BR. The counterions and water molecules (w5, w8, and w10) form a hydrogen bond pentamer, which stabilizes the RSB and the extracellular part of the retinal binding pocket. The counterions take the same positions as the corresponding amino acids of *Hs*BR, and as in *Hs*BR, are additionally stabilized by T143 and S97 residues. By contrast, Gloeobacter rhodopsin (GR) and other proteorhodopsins (PRs) exhibit a different conformation of the RSB region^43^. In the GR structure, D121 (counterpart to D85 in *Hs*BR, the primary proton acceptor) forms a hydrogen bond with H57^44^. This configuration of the counterions was shown to cause pH dependence of the photocycle due to deprotonation of the H57 residue at pH higher than physiological^45^ (Supplementary Figure 5). In archaeal pumping rhodopsins, the proton release is mediated by a glutamate pair (E194–E204 in *Hs*BR). In LR and other eukaryotic proton pumps, the release group has a similar construction. The E258 residue interacts with D248 that equivalently substitutes E204 of *Hs*BR in a configuration similar to that of *Hs*BR. By contrast, the bacterial proton-release group consists of only one negatively charged residue, the sole glutamate in TM5 or TM6 (Supplementary Figures 6 and 7). A common feature of bacterial proton pumps is that they contain a water-accessible cavity that is connected with the extracellular bulk and protrudes up to R136 (Fig. 2a). By contrast, in both archaeal and eukaryotic proton-pumping rhodopsins, extracellular cavities are separated by a negatively charged pair of residues (E194–E204 in *Hs*BR and D248–E258 in LR) into two parts, one of which is open to the bulk (Fig. 2a). The proton-releasing moiety of PRs consists of the sole glutamate residue in TM5. The configuration of this part is conserved in the entire family of bacterial proton-pumping rhodopsins (Supplementary Figure 7). The extracellular part of the protein is fully accessible from the bulk for the water molecules up to the arginine residue (R76 in *Med12*BPR), similarly to LR.

The intracellular portion of LR is structurally nearly identical to that of *Hs*BR (Fig. 2b). In particular, reprotonation occurs through the D150 residue, presumably, followed by a synchronized movement of L147 and T143 residues^46^. In *Hs*BR, the corresponding residues are D96, L93, and T90, respectively. In the GR structure, the residues responsible for the proton uptake are E132, Q129, and T125. This configuration results in nearly complete accessibility of the proton donor E132 to the cytoplasmic bulk (Supplementary Figure 8). In other proteorhodopsins, the glutamate is often replaced by lysine, causing an absorbance shift effect^47^.

### Photocycle of LR proton pump

**Fig. 3:**
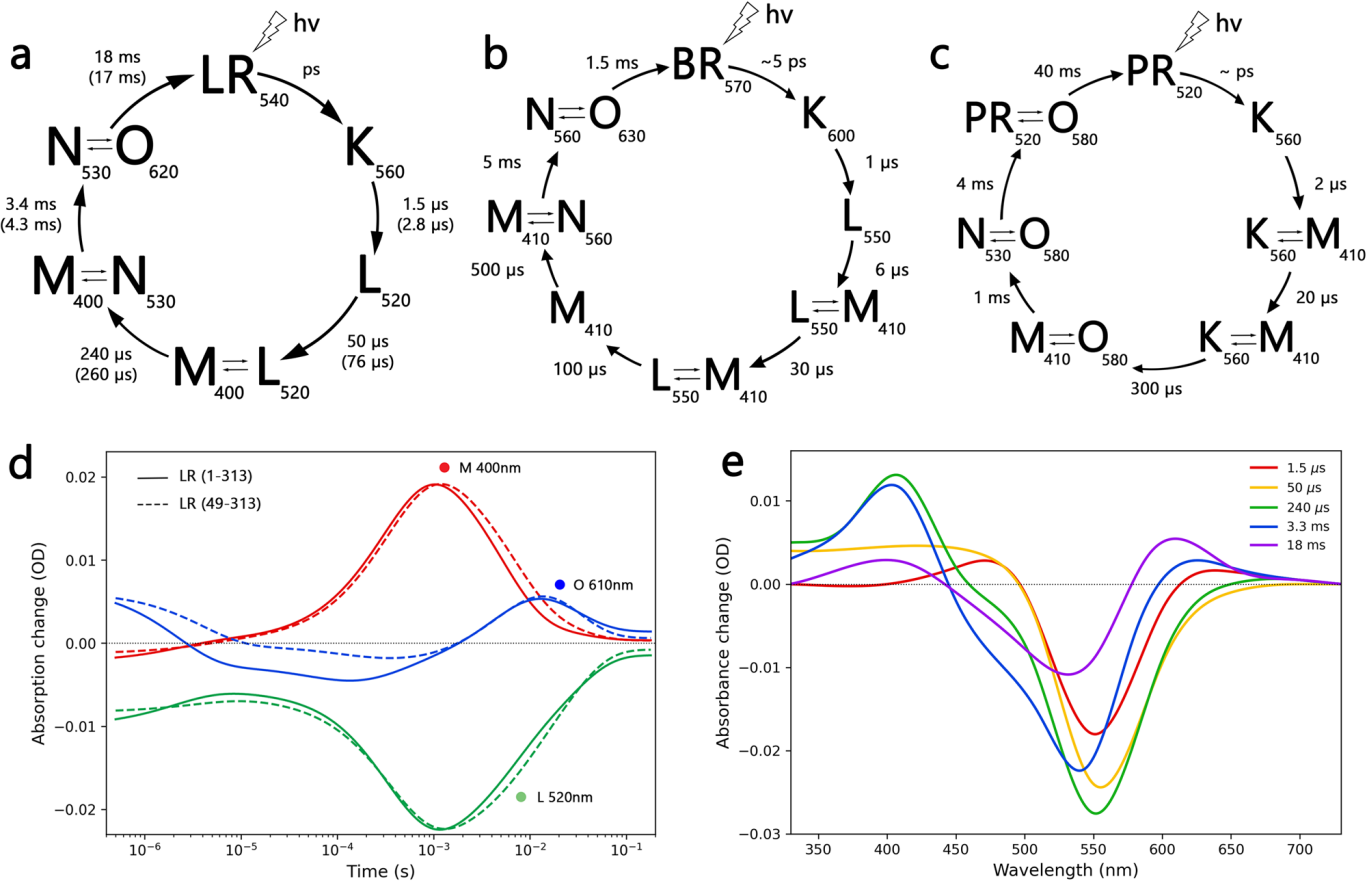
Spectroscopic characterization of LR. Schematic representation of (**a**) LR, (**b**) *Hs*BR, and (**c**) GPR photocycle kinetics^50,54^. Lifetimes of LR 1–313 and LR 49–313 are indicated in brackets and without brackets, respectively. The retinal absorbance maximum of the intermediate states is indicated. **d** Time traces of the absorption changes of LR 1–313 and LR 49–313 at 400, 520, and 610 nm wavelengths. **e** Differential absorption spectra of five intermediates of the LR 1–313 photocycle. Full comparison between LR construct photocycle kinetics can be found in Supplementary Figure 9.

The photocycle kinetics of *Hs*BR and LR are closely similar as suggested by the high structural similarity and are likely to undergo similar structural rearrangements under light illumination^48^ (Fig. 3). Both full-length and truncated constructs of LR in DDM micelles exhibit almost similar relaxation time that suggests that N-terminus has little impact on protein photoactivation (Supplementary Figure 9). After photoactivation, the LR photocycle starts with a short-living K state followed by blue-shifted L- and M states associated with proton release in archaeal proton pumps^49^. Next, the protein relaxation into the ground state occurs through N- and O states that complete the photocycle^50–53^. Consistent with RSB configuration difference, PR photocycle, and in the particular photocycle of green-absorbing proteorhodopsin (GPR) has 4 photointermediate states under neutral pH, lacking an apparent L state^54^. Consistent with structural similarity, the photocycle of LR and *Hs*BR demonstrates nearly similar photocycle composition that further supports a close relation between LR and archaeal proton pump *Hs*BR.

### LR oligomeric state

**Fig. 4:**
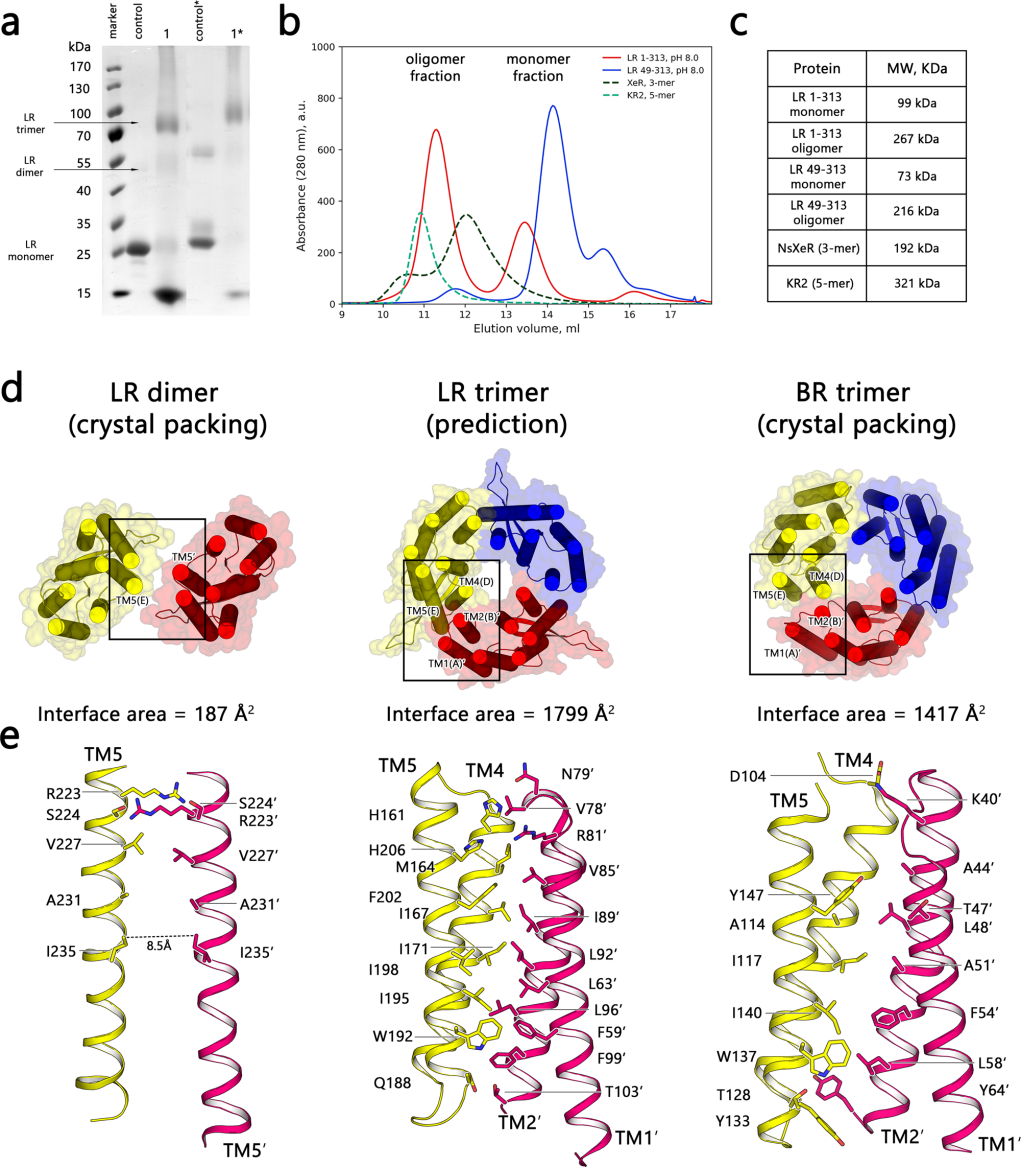
LR oligomerization analysis. **a** Glutaraldehyde cross-linking of LR 1–313 and LR 49–313. Gradient SDS–PAGE 18.5–8%. Lanes 1 and 1* contain LR 1–313 and LR 49–313 samples treated with glutaraldehyde vapor for 30 min correspondingly. LR samples not treated with glutaraldehyde were used as a control. **b** Size-exclusion chromatography profiles of LR 1–313 and LR 49–313 protein used for crystallization trials. Elution profiles of *Ns*XeR and KR2 proteins are shown as examples of trimeric and pentameric proteins with similar SEC experiments. Full details on the expression and purification of those proteins can be found in^5,6^. **c** The estimated molecular weight of different rhodopsin fractions obtained during the SEC experiment using standard calibration proteins. Full details on cross-linking and SEC calibration can be found in Supplementary Figure 3. **d** Extended comparison of multimeric states of LR and *Hs*BR. LR dimer and *Hs*BR trimer correspond to the crystal-packing multimeric state. LR trimeric state was calculated using Homomer server^60^ with archaeorhodopsin-2 reference model (PDB: 3WQJ). **e** Magnified view of an interprotein interaction between monomers in the multimeric state. The average interaction surface area is indicated for all structures.

Almost all microbial rhodopsins exist as oligomers in the native lipid environment. The oligomeric state was previously shown to contribute to protein stability^55,56^ and in some cases to function^57^. Besides that, the stoichiometry of the fundamental oligomer of microbial rhodopsins was recently found to correlate with the phylogenetic origin of rhodopsins^58^. Even though LR forms dimers in the crystal lattice (Supplementary Figure 1), the monomer–monomer interface was surprisingly low (187 Å^2^)^59^. To assess oligomeric states of LR rhodopsin in a solution, we performed glutaraldehyde cross-linking of LR followed by SDS-PAGE analysis (Fig. 4a). This method was previously used in combination with size-exclusion chromatography (SEC) to confirm pentameric assembly of viral rhodopsin OLPVRII^17^, so we believe that combination of cross-linking with SEC provides sufficient reliability for the determination of LR protein oligomerization. For both LR 1–313 and LR 49–313 we observed bands corresponding to oligomers with stoichiometry 3 and lower, which suggests trimeric state as predominant in DDM micelles (Fig. 4a, Extended Fig. 10). Moreover, size-exclusion chromatography (SEC) profiles of LR 1–313 and LR 49–313 in DDM micelles demonstrate the presence of only one oligomeric state, which is likely trimeric. At pH 8.0, the calculated molar masses from the SEC retention volume of LR 1–313 and LR 49–313 peaks were 99 kDa/267 kDa and 73 kDa/216 kDa, respectively (Fig. 4b). Given that the empty DDM micelle size is ~50 kDa, pentameric KR2 and trimeric *Ns*XeR elutes at 321 and 192 kDa respectively (Fig. 4c). Therefore, it is likely that LR in DDM micelles exist in monomeric and trimeric forms only. It is also noteworthy that truncation of N-terminus changes the ratio between monomers and trimers from ~2:5 for LR 1–313 to ~40:1 for LR 49–313 and thus impacts on detergent resistance of the protein.

Using template-based modeling GalaxyHomomer server, we proposed the trimeric state of LR using monomer template-based on archaeorhodopsin-2 model (PDB: 3WQJ)^60^. LR trimer has a higher monomer–monomer interface area (1799 Å^2^) and therefore is a likely major oligomeric form of LR rhodopsin (Fig. 4d). Besides that, protomers in both LR and *Hs*BR trimers interact within TM2’–TM4 helices (Fig. 4e), that further highlights the similarity between LR and HsBR. It should also be noted that despite the similarity of ECL1 of LR and ICL1 of heliorhodopsins, heliorhodopsins employ different dimer configuration with a much higher interface surface area (Supplementary Figure 11)

### Structural roles of ECL1 and the N-terminal domain

**Fig. 5:**
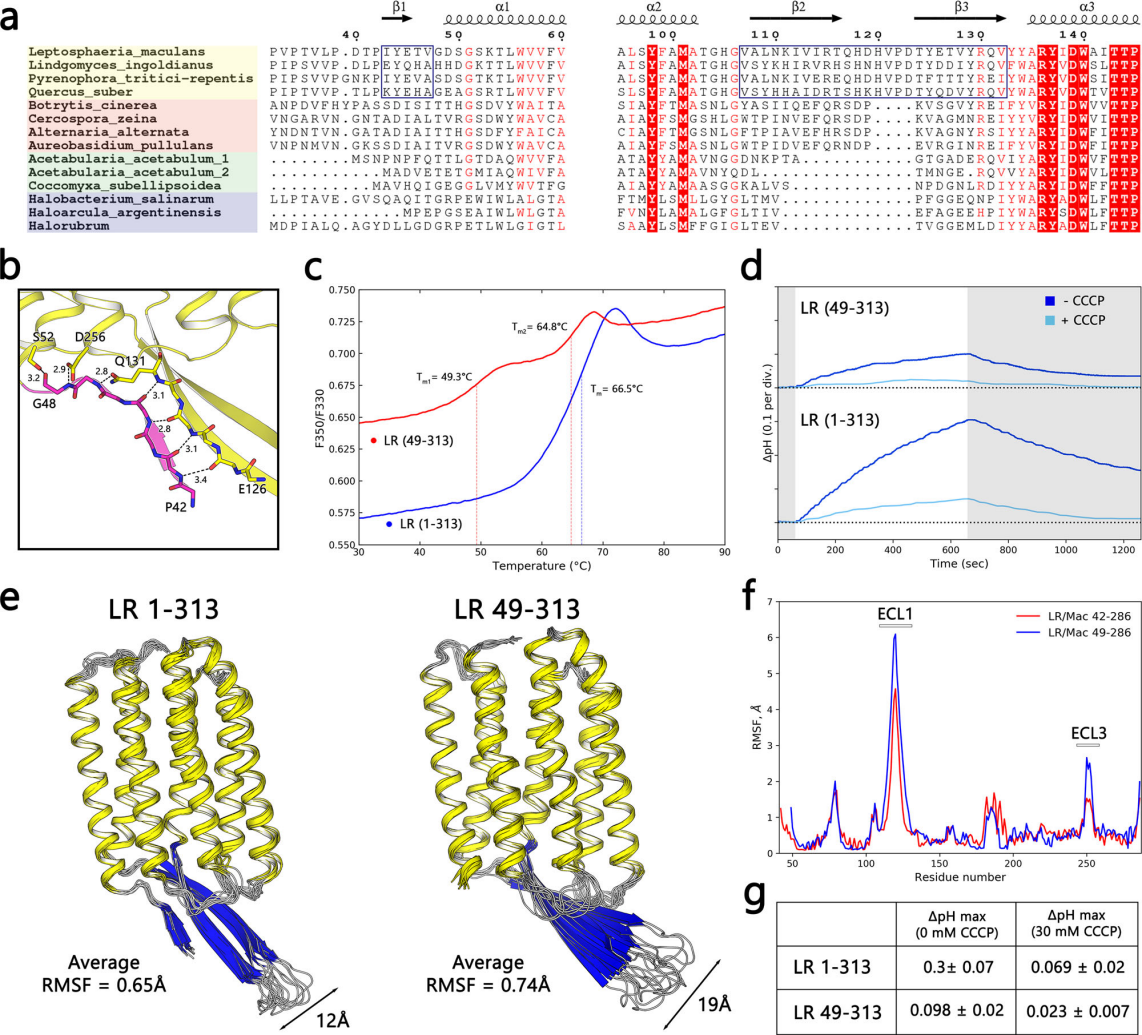
Influence of N-terminal domain on LR structural stability and function. **a** Sequence alignment of BC loops and N-terminal domains of selected fungal (yellow, orange), algal (green), and archaeal (purple) proton pumps. Elongated BC-loop and N-terminal beta-strand variants are additionally indicated with blue frames. Highly conservative amino acids are highlighted in red. **b** Magnified view of LR N-terminal domain region, residues 42–48 subjected for truncation are colored magenta. Seven hydrogen bonds involved in ECL1 stabilization are indicated. **c** Thermostability of LR 1–313 and LR 49–313 measured with Nano-DSF assay at pH 7.0 in 0.05% DDM (*n* = 1). Inflection points and their corresponding temperatures are indicated with dash lines. **d** Representative examples of pH change upon illumination in liposome suspension with reconstituted LR 1–313 and LR 49–313 before (dark blue) and after (light blue) addition of 10 μM of CCCP. **e** Protein-structure flexibility trajectories (*n* = 10) of LR 42–286 and LR 49–286 calculated using CABS-flex 2.0 server^65^. For LR 49–286 initial model residues, 42–48 were removed from LR 42-286 crystal structure. Average root mean square fluctuations (RMSF) are shown for both models. **f** Averaged RMSF profiles of LR 42–286 and LR 49–286 models. The most disordered regions (ECL1 and ECL3) are highlighted. **g** Comparison between maximum pH change after 10 min under light illumination of LR constructs. Values are shown as mean ± SD, *n* = 3 for both constructs. The same sample was measured repeatedly.

We showed that the LR core (membrane part) of the protein is strikingly similar to the core of *Hs*BR and, in general, to the corresponding structures of other archaeal rhodopsin proton pumps as expected of core parts of highly conserved homologous proteins. By contrast, substantial structural variation could be expected to exist in the N- and C-terminus regions. To assess the role of the N-terminal domain in the structural stability of the protein, we searched protein sequence databases for homologs of LR using BLASTP^61^ and identified multiple opsins with high sequence similarity to LR. Despite the overall high similarity, we identified at least two subgroups among fungal rhodopsins that follow different patterns in the extracellular side of the protein. The first subgroup contained an elongated ECL1 domain, and a short beta-strand bears the N-terminus, whereas the second subgroup lacked both these features (Fig. 5a, Supplementary Figure 12). Secondary-structure prediction with RAPTORX^62^ suggests the presence of an additional beta-strand near the N-terminus of LR, but not in subgroup 2 of fungal rhodopsins or other light-driven proton pumps. Therefore, the elongated ECL1 domain and the N-terminal beta-strand appear to be signatures of the subfamily of fungal rhodopsins that includes LR rhodopsin. Consistent with the prediction, the LR crystal structure contained 8 residues from the N-terminal region (residues 42–49), that aligned with ECL1 and belonged to a 3-stranded antiparallel β-sheet. This N-terminal β-strand strongly interacts with the ECL1 loop and is directly involved in its stabilization through at least 7 hydrogen bonds. In addition, the N-terminus of LR is connected with the TM α-helices via the V47–S52 and G48–D256 interactions (Fig. 5b). To verify the effect of the N-terminal truncation, we measured the thermal stability of the LR protein constructs with a nano-DSF method under different pH and detergent concentrations^63^ (Supplementary Figure 13). The F350/330 ratio of truncated LR showed two inflection points (corresponding to protein-unfolding temperatures) at 49.3 and 64.8 **°**C, whereas the full-length LR demonstrated a single inflection point at 66.5 **°**C (Fig. 5c).

To assess the contribution of the N-terminal domain^64^ to protein stabilization we used CABS-flex 2.0 coarse-grain protein modeling server^65^. We used two models, full-length LR structure (resolved residues 42–286) and LR structure without the N-terminal region (residues 49–286), that represent LR constructs used for functional tests (Fig. 5e). Consistent with previous observations, the average root mean square fluctuation (RMSF) of LR 49–286 (0.74 Å) was higher than the average RMSF of LR 42–286 (0.65 Å). In particular, the highest destabilization was predicted for the ECL1 and ECL3 domains that directly interact with the N-terminal domain in the crystal structure. Given that the F350/330 ratio represents a change in tryptophan fluorescence, the additional melting point at 49.3 **°**C is likely to be caused by tryptophans on the extracellular side of the protein (W56, W190, W192, and W244). Increased flexibility of TM1, ECL1, and ECL3 domains can impact hydration and orientation of tryptophans on the extracellular side, with surprisingly little influence on protein kinetics.

### Proton translocation experiments

To estimate the proton-pumping activity of the full-length and truncated proteins, we performed ion translocation experiments using POPC:POPS proteoliposomes (see “Methods” for details). Under weak acidic conditions (pH 6.5, 100 mM NaCl), the full-length protein demonstrated a substantially higher proton-pumping activity than the truncated derivative, 0.3 pH units for LR 1–313 against 0.1 for LR 49–313 (Fig. 5d, g).

A possible explanation of such a difference in proton pumping activity of two proteins with nearly similar photocycle is protein orientation in liposomes. Membrane protein orientation generally follows the “positive-inside” rule^66,67^. Truncation of a slightly negatively charged N-terminus (D4, E7, E8, and D40) substantially altered the charge distribution of the extracellular part of the LR protein. Because extracellular parts of membrane proteins are more negatively charged than intracellular parts, such removal should influence protein orientation in both cell membranes and liposomes. Besides, protein orientation might be also influenced by decreased stability of the extracellular portion of the truncated protein.

### Structure-based phylogenetic analysis of light-driven proton pumps

**Fig. 6:**
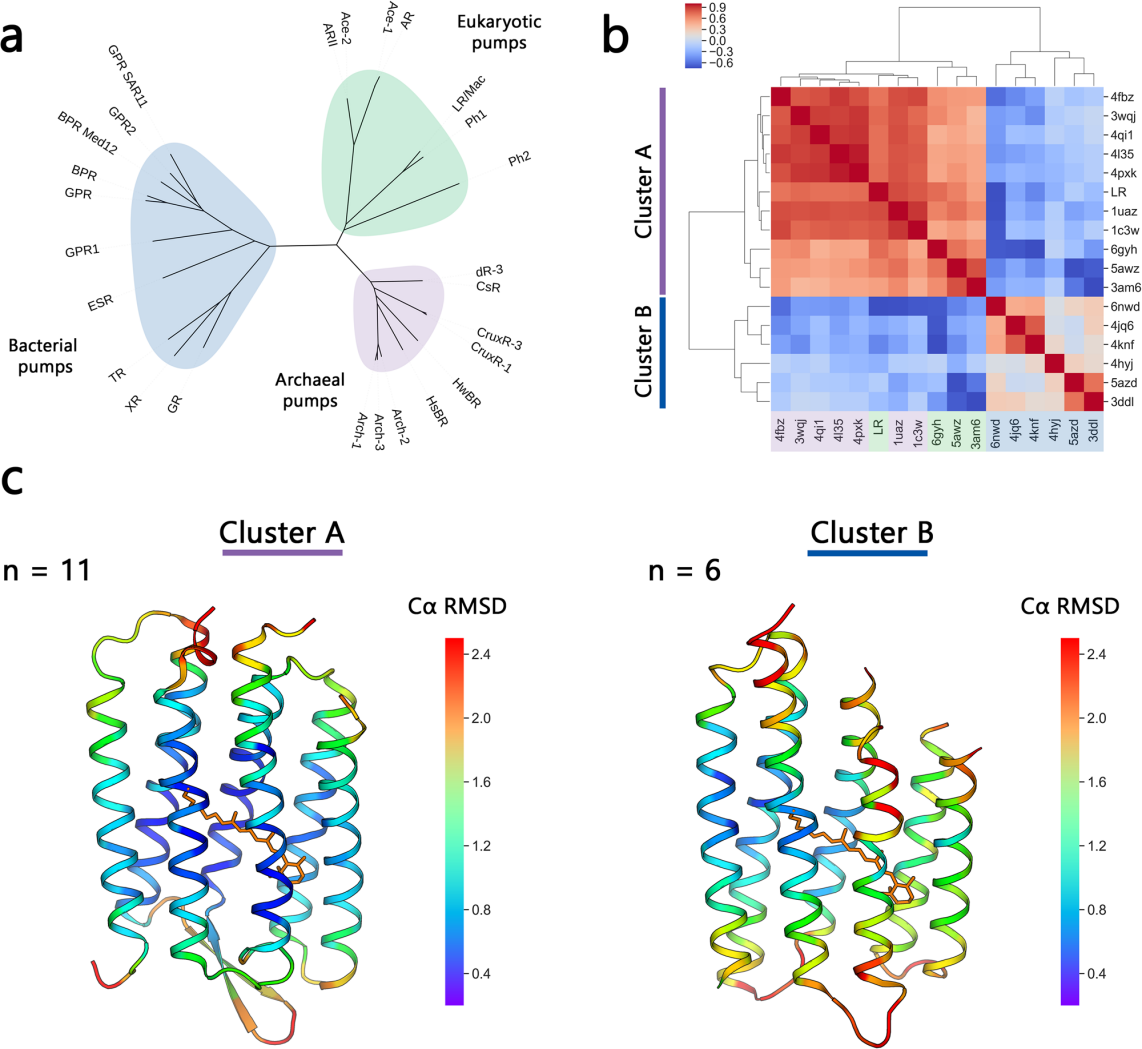
Structure-based phylogenetic analysis of light-driven proton pumps. **a** Phylogenetic tree of selected rhodopsins with H^+^-pumping activity. **b** Correlation value heatmap of pairwise RMSD of known structures of H^+^-pumping rhodopsins, including LR. The {indicated} PDB IDs of the structures are arranged following hierarchical clustering of the correlation values. **c** Structures of *Hs*BR (PDB ID: 1C3W) and *Med12*BPR (PDB ID: 4QJ6) were used as “reference” structures for *cluster A* (left) and *cluster B* (right). C*α* atom-position conservation within each cluster as the corresponding C*α*_*RMSD*_ value, determined as described in Methods, is indicated by color for each residue according to the presented color bar. The number of the proteins in each cluster is additionally indicated by *n* value.

Different aspects of the evolution of microbial rhodopsins have been addressed in detail, featuring both large datasets^68^ and rhodopsins with putative functions^24,69–71^. However, all the conclusions in these studies are derived from sequence-based phylogenetic analysis. To address different perspectives of the discussion, we performed a structure-based clustering of the light-driven outward proton pumps using 16 structures available from the PDB (see Methods for details). To this end, we superimposed all structures of proton pumps and calculated the pairwise RMSD values. We used these values to cluster the rhodopsins with a hierarchical clustering algorithm (Fig. 6). Notably, and in agreement with both the detailed structural comparisons described here and the previous phylogenetic analyses^24,68^, the proton-pumping rhodopsins from archaea and eukaryote confidently clustered together, to the exclusion of the structurally distinct bacterial rhodopsins (Fig. 6b). Rhodopsin genes appear to be subject to extensive horizontal gene transfer, resulting in mixed branches in phylogenetic trees and complicating inferences of common ancestry^72,73^. Nevertheless, the high structural similarity between archaeal and eukaryotic proton pumps including the conformation of key functional regions (Fig. 6c), such as the retinal binding pocket and the regions involved in proton release and proton uptake, along with functional similarities (pH dependence of the photocycle, the intermediate states), presents strong evidence of an archaeal origin of eukaryotic proton-pumping rhodopsins (and, most likely, all other eukaryotic rhodopsins as well). Thus, rhodopsins appear to belong to a small set of genes, apart from the core components of the translation, transcription, and replication systems, that have been inherited by the protoeukaryotes from their archaeal ancestors.

## Methods

### Phylogenetic analysis

In total, 28 sequences of rhodopsins with H^+^-pumping activity were aligned using MUSCLE. Phylogenetic reconstruction was conducted by maximum likelihood (ML) using PhyML with the following parameters: Jones–Taylor–Thornton model, SH-like approximate likelihood-ratio test, and estimated gamma-distribution parameter. The following rhodopsins were used for phylogenetic tree construction and sequence alignment:

*Hs*BR, Bacteriorhodopsin from Archaea (*Halobacterium salinarum;* P02945); HwBR/MR, Midrhodopsin from Eubacteria (*Haloquadratum walsbyi* DSM 16790*,* Q18DH8); aR-1, Archaerhodopsin-1 from Archaea (*Halorubrum chaoviator*, P69051); aR-2, Archaerhodopsin-2 from Archaea (*Halobacterium sp*. AUS-2, P29563), aR-3, Archaerhodopsin-3 from Archaea (*Halorubrum sadomense*, P96787); cR-1, Cruxrhodopsin-1 from Archaea (*Haloarcula argentinensis*, Q57101); cR-3, Cruxrhodopsin-3 from Archaea (*Haloarcula vallismortis*, P94854); dR-3, Deltarhodopsin-3 from (*Haloterrigena thermotolerans*, I4DST7); LR, Leptosphaeria Rhodopsin from Eukaryote (*Leptosphaeria maculans*, Q9HGT7); ARII/Ace2, Acetabularia Rhodopsin II from Eukaryote (*Acetabularia acetabulum*, G1K3Q0); AR, Acetabularia Rhodopsin from Eukaryote (*Acetabularia acetabulum*, Q1AJZ3); CsR, Coccomyxa subellipsoidea rhodopsin from Eukaryote (*Coccomyxa subellipsoidea* C-169, I0YUS5); Ph2, Phaeosphaeria Rhodopsin 2 from Eukaryote (*Phaeosphaeria nodorum* SN15, Q0V5A7); Ph1; Phaeosphaeria Rhodopsin 1 from Eukaryote (*Phaeosphaeria nodorum* SN15, Q0V6M3); ESR, Exiguobacterium sibiricum rhodopsin from Eubacteria (*Exiguobacterium sibiricum* DSM 17290, B1YFV8); *Med12*, proteorhodopsin from uncultured bacterium (*Med12*, Q4PP54); HOT75, Blue-light absorbing proteorhodopsin from uncultured bacterium (HOT 75m4, Q9AFF7); XR, Xanthorhodopsin from Eubacteria (*Salinibacter ruber* DSM 13855/M31, Q2S2F8); TR, Thermophilic Rhodopsin from Eubacteria (*Thermus thermophilus* JL-18, H9ZSC3); gPR, Green-light absorbing proteorhodopsin from Eubacteria (*Gamma-proteobacterium EBAC31A08*, Q9F7P4); MacR, Mac Rhodopsin from Eubacteria (*Candidatus Actinomarina minuta*, S5DM51); PR from *O.marina*, Oxyrrhis marina rhodopsin (*Oxyrrhis marina (Dinoflagellate)*, A7WQE3); GR, Gloeobacter Rhodopsin from Eubacteria (*Gloeobacter violaceus* ATCC 29082/PCC 7421, Q7NP59), GPR1, Green-absorbing Proteorhodopsin from Eubacteria (*Dokdonia donghaensis* MED134, EAQ40507); GPR2, Green-absorbing Proteorhodopsin from Eubacteria (*Vibrio sp*. AND4, ZP_02194911.1); GPR3, Green-absorbing Proteorhodopsin from Eubacteria (*Candidatus Pelagibacter ubique* HTCC1062, “SAR11” group, Q4FMZ3); BPR, Blue-absorbing Proteorhodopsin from Eubacteria (*Photobacterium sp*. LC1-200, BAL68143); NM-R1, Nonlabens marinus proteorhodopsin from Eubacteria (*Nonlabens marinus* S1-08, W8VZ92).

### Structure alignment

For structure alignment, 17 known atomic structure models of H+ -pumping rhodopsins, including LR, were compared pairwise by RMSD values calculated with the PyMOL *align* function with parameter cycles = 2 and cutoff = 1.5. After hierarchical clustering of the RMSD correlation values, the analyzed structures were divided into two clusters - “archaeal” and “bacterial”. Then the structure of the reference archaeal pump *Hs*BR was aligned to each one from the “archaeal” cluster and the structure of the reference *Med12*BPR was aligned to each one from the “bacterial” cluster. To represent the conservation of Cα atom positions within the clusters, for every Cα atom of the reference structure, distance to the nearest Cα atom of the respective aligned structure was measured. The measurement was repeated for every aligned structure. RMSD of the distances corresponding to each Cα atom of each reference structure, Cα_RMSD_, was used as a measure of the Cα position conservation.

### Cloning

The genes encoding LR (1–313 aa and 49–313 aa) from *Leptosphaeria maculans* (UniProt Q9HGT7) were synthesized de novo. The nucleotide sequences were optimized for *Leishmania tarentolae* expression with GeneOptimizer software (Thermo Fisher Scientific). Both genes in fusion with the C-terminal polyhistidine tags (H6 and H9) were introduced into the integrative inducible expression vector *pLEXSY_I-blecherry3* (Jena Bioscience, Germany) through the BglII and NotI restriction sites. Plasmid vectors, optimized genes, and primers used for PCR amplification can be found in Supplementary Tables 1–3.

### LEXSY expression, solubilization, and purification

The proteins were expressed as described previously^36^. The *Leishmania tarentolae* cells of the strain LEXSY host T7–TR (Jena Bioscience) were transformed with the LR 1–313 and LR 49–313 expression plasmids linearized by the SwaI restriction enzyme. After the clonal selection, the transformed cells were grown at 26 °C in the dark in shaking baffled flasks in the Brain-Heart-Infusion Broth (Carl Roth, Germany) supplemented with 5 mg ml^−1^ Hemin, 50 U ml^−1^ penicillin, and 50 mg ml^−1^ streptomycin (AppliChem). When OD_600_ = 1.0 was reached, 10 mg ml^−1^ tetracycline was added, for LR 1-313 also, 10 μM all-trans-retinal (Sigma-Aldrich) was added, and incubation continued for a further 24 h. The collected cells were disrupted in an M-110P Lab Homogenizer (Microfluidics) at 10,000 psi in a buffer containing 50 mM NaH_2_PO_4_/Na_2_HPO_4_, pH 7.6, 0.1 M NaCl, 10% glycerol, 1 mM EDTA, 2 mM 6-aminohexanoic acid (AppliChem), 50 mg L^−1^ DNase I (Sigma-Aldrich), and complete protease inhibitor cocktail (Roche). The membrane fraction of the cell lysate was isolated by ultracentrifugation at 120,000 g for 1 h at 4 °C. The pellet was resuspended in the same buffer but without DNase I and stirred for 30 min at 4 °C. The ultracentrifugation step was repeated. Finally, the membranes were resuspended in the solubilization buffer containing 20 mM HEPES, pH 8.0, 0.2 M NaCl, cOmplete, 1% n-dodecyl-β-D-maltoside (Cube Biotech), and 20 μM for LR 1–313 and 5 μM for LR 49–313 all-trans-retinal and stirred overnight for solubilization. The insoluble fraction was removed by ultracentrifugation at 120,000 g for 1 h at 4 °C. The supernatant was loaded on a Ni-NTA resin (Cube Biotech), and LR was eluted in a buffer containing 20 mM HEPES, pH 7.5, 0.2 M NaCl, 0.25 M L-Histidine (AppliChem), 0.1 mM phenylmethanesulfonyl fluoride (PMSF), 2 mM 6-aminohexanoic acid, cOmplete, and 0.1% n-dodecyl β-D-maltoside. The eluate was subjected to size-exclusion chromatography on a Superdex 200 Increase 10/300 GL column (GE Healthcare Life Sciences) in a buffer containing 10 mM NaH_2_PO_4_/Na_2_HPO_4_, pH 6.5, 0.2 M NaCl, 1 mM EDTA, 2 mM 6-aminohexanoic acid, cOmplete, 0.1 mM phenylmethanesulfonyl fluoride and 0.05% n-Dodecyl β-D-maltoside. Protein-containing fractions with an A_280_/A_540_ absorbance ratio of about 1.5 were pooled and concentrated to 30–40 mg ml^−1^ for crystallization. An average yield for LEXSY-optimized constructs of LR 1–313 and LR 49–313 was 20 mg and 10 mg from 1 liter of culture, respectively.

### Incorporation of the protein into liposomes

Phospholipids 1-palmitoyl-2-oleoyl-glycero-3-phosphocholine and 1-palmitoyl-2-oleoyl-sn-glycero-3-phospho-L-serine at a ratio 4:1 (wt wt^−1^) (POPC:POPS) were dissolved in CHCl_3_ (chloroform ultrapure, Applichem Panreac) and dried under a stream of N_2_ in a glass vial. The solvent was removed by overnight incubation under vacuum. The dried lipids were resuspended in 4% (w v^−1^) sodium cholate. The mixture was clarified by sonication at 4 °C and LR was added at a protein:lipid ratio of 1:20 (w w^−1^). The detergent was removed by the addition of detergent-absorbing beads (Amberlite XAD 2, Supelco) and incubation at 4 °C for 2 days. The 1.5 ml of liposome mixture was dialyzed against 100 mM NaCl (pH 8.0) buffer at 4 °C for 18 h (two 1.5 L changes) to adjust for the desired pH.

### Measurement of proton translocation activity in proteoliposomes

The measurements were performed on 1.5 ml of stirred liposome suspension at 4 °C. LR-containing liposomes were prepared following the protocol described above. Liposomes were illuminated for 10 minutes with a halogen lamp (Intralux 5000-1, VOLPI) and then were kept in the dark for another 10 min. Changes in pH were monitored with a pH meter (LAB 850, Schott Instruments). Measurements were repeated in the presence of 30 μM of carbonyl cyanide m-chlorophenyl hydrazine (CCCP, Sigma-Aldrich) under similar conditions.

### Nano-DSF measurements

Nano-DSF measurements were performed using a Prometheus NT.48 instrument (NanoTemper Technologies, Germany). In total, 6 samples for both full-length and truncated LR were made, having pH 6.0, 7.0, or 8.0 and DDM concentration of 0.05% and 1%. First, samples with purified LR at a concentration of 1 mg ml^−1^ were dialyzed against 200 mM NaCl, 0.05% DDM buffer for 18 h at 4 °С. After that, the protein was introduced into 50 mM Na_2_HPO_4_/NaH_2_PO_4_, 200 mM NaCl buffer with the desired pH, and incubated at 4 °C for another 18 h. About 10 μL of each sample was loaded into UV capillaries (NanoTemper Technologies). The temperature gradient was set at 1 °C min^−1^ in a range from 15 to 98 °C. Protein unfolding was observed by following the change in tryptophan fluorescence at emission wavelengths of 330 and 350 nm. The ratio between the emission intensities at 350 and 330 nm (F350/F330) was used to track the structural changes with increasing temperature. Melting-point temperatures (T_m_) were calculated using the peaks in the first derivative of the signal data.

### Time-resolved absorption spectroscopy

The laser-flash photolysis setup was similar to that described by Chizhov and co-workers^50^. The excitation/detection systems were composed as such: Brilliant B laser with OPO Rainbow (Quantel Inc.) was used, providing pulses of 4-ns duration at 530-nm wavelength and an energy of 2 mJ per pulse. Samples (5 × 5 mm spectroscopic quartz cuvette; Hellma, Germany) were placed in a thermostated house between two collimated and mechanically coupled monochromators (LOT MSH150). The probing light (xenon arc lamp, 75 W, Hamamatsu) passed the first monochromator sample and arrived after a second monochromator at a photomultiplier tube (PMT) detector (R12829, Hamamatsu). The current-to-voltage converter of the PMT determines the time resolution of the measurement system of ca. 50 ns (measured as an apparent pulse width of the 5-ns laser pulse). Two digital oscilloscopes (Keysight DSO-x4022A, 2 units, 25 kilobytes of buffer memory per channel, respectively) were used to record the traces of transient transmission changes in two overlapping time windows. The maximal digitizing rate was 10 ns per data point. Transient absorption changes were recorded from 10 ns after the laser pulses until the full completion of the phototransformation. At each wavelength, 25 laser pulses were averaged to improve the signal-to-noise ratio. The quasi logarithmic data compression reduced the initial number of data points per trace (~32,000) to ~850 points evenly distributed in a log time scale giving ~100 points per time decade. The wavelengths were varied from 330 to 700 nm in steps of 10 nm using a computer-controlled step motor. The absorption spectra of the samples were measured before and after each experiment on a standard spectrophotometer (Avantes Avaspec 2048 L).

### Data treatment and global fit analysis

Each data set was independently analyzed using the global multiexponential nonlinear least-squares fitting program MEXFIT^50^. The number of exponential components was incremented until the SD of weighted residuals did not further improve. After establishing the apparent rate constants and their assignment to the internal irreversible transitions of a single chain of relaxation processes, the amplitude spectra of exponents were transformed to the difference spectra of the corresponding intermediates in respect to the spectrum of the final state. Subsequently, the absolute absorption spectra of states were determined by adding the difference spectra divided by the fraction of converted molecules to the spectra of the final states. Criteria for the determination of the fraction value were the absence of negative absorbances and contributions from the initial state to the calculated spectra of the final state.

### Crystallization

The crystals were grown with an *in meso* approach^74–76^, similar to that used in our previous works^7,8^. The solubilized protein (40 mg ml^−1^) in the crystallization buffer was mixed with premelted at 42 °C monoolein (MO, Nu-Chek Prep) in 3:2 ratio (lipid: protein) to form a lipidic mesophase. The mesophase was homogenized in coupled syringes (Hamilton) by transferring the mesophase from one syringe to another until a homogeneous and gel-like material was formed^77^. About 150 nl drops of a protein–mesophase mixture were spotted on a 96-well LCP glass sandwich plate (Marienfeld) and overlaid with 400 nL of a precipitant solution using the NT8 crystallization robot (Formulatrix). The best crystals were obtained with a protein concentration of 20 mg ml^−1^ and 100 mM HEPES, pH 7.0, 4% PEG 3350, 1 M sodium malonate pH 7.0. The crystals were grown at 22 °C and appeared in 1–4 weeks. The needle-like crystals grew to 100 μm in size. Once crystals reached their final size, crystallization wells were opened as described previously^78^, and drops containing the protein–mesophase mixture were covered with 100 μl of the respective precipitant solution. All crystals were harvested using micromounts (MiTeGen), flash-cooled, and stored in liquid nitrogen before data collection.

### Data collection

X-ray diffraction data were collected at ID30b beamline at ESRF, Grenoble, France at 100 K with a PILATUS3 6 M (Dectris) detector. Diffraction images were processed with XDS software and initially scaled using Aimless from the CCP4 suite. The analysis of the anisotropy indicated that the data are anisotropic; therefore, it was also treated using Staraniso server^79^. The crystallographic data statistics are shown in Table 1.

### Structure refinement

Reference model (*Hs*bR, PDB 1C3W) was used for molecular replacement using Molrep from the CCP4 suite. The initial phases were successfully obtained in the P212121 space group. The initial model was iteratively refined using REFMAC5^80^, PHENIX^81^, and Coot^82^. Anisotropically treated data on LR improved the quality of the electron-density maps compared with the isotropically treated data. Therefore, the data from the Staraniso server were used to build the final model. However, the completeness of the anisotropically treated data is low (40% at the highest-resolution shell). Consequently, we refined the final model against the isotropically treated data to obtain the most reliable structure and the corresponding electron-density maps. Importantly, R-free flags were the same for both anisotropically- and isotropically- treated data. The final R work/R free values and refinement statistics are shown in Table 1. The R work /R free values are unexpectedly high for the 2.2 Å resolution, which is most likely due to the presence of a high level of translational non-crystallographic symmetry (tNCS) as indicated by Xtriage software from PHENIX

### Reporting summary

Further information on research design is available in the Nature Research Reporting Summary linked to this article.

## Supplementary information

Supplementary information

Description of Supplementary Files

Supplementary Data 1

Reporting Summary

## Data Availability

The protein coordinates and atomic structure factors have been deposited in the Protein Data Bank (PDB) under accession number 7BMH (LR). Source data are provided as Supplementary Data 1. All other data are available from the corresponding author upon reasonable request.
