# Supplementary material for: Structure-based insights into evolution of rhodopsins
Source: Commun Biol. 2021 Jun 30;4:821. doi: 10.1038/s42003-021-02326-4 (PMC8245419; doi:10.1038/s42003-021-02326-4)
Supplement: Supplementary file 1 — Supplementary information [file 42003_2021_2326_MOESM1_ESM.pdf]

Supplementary Information.

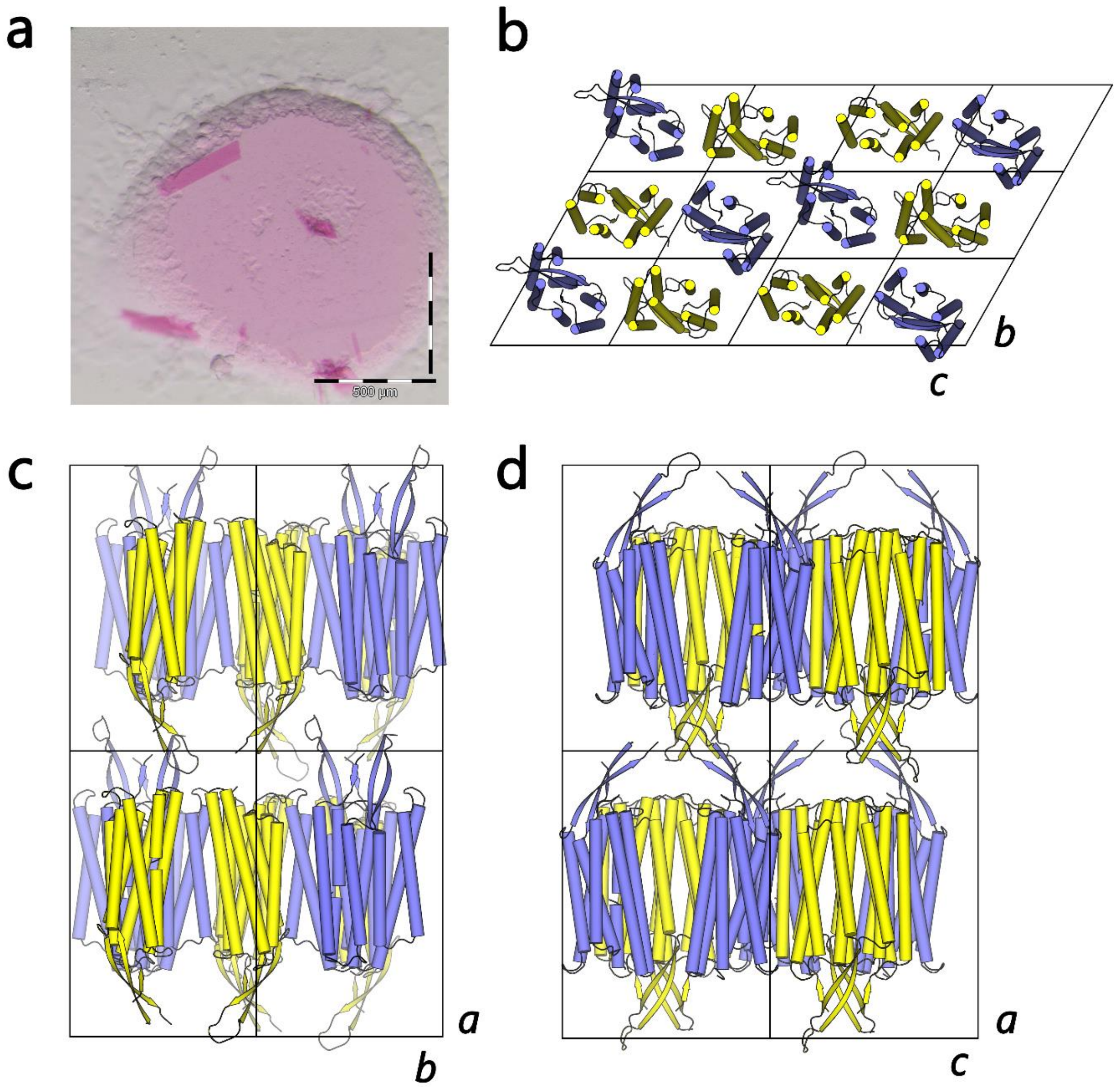

Supplementary Figure 1. Crystal packing of LR. (a) Example of the LR crystal grown using *in meso* approach. (b)-(d) LR symmetry units in three projections with the corresponding axis. α-helices are presented as cylinders.

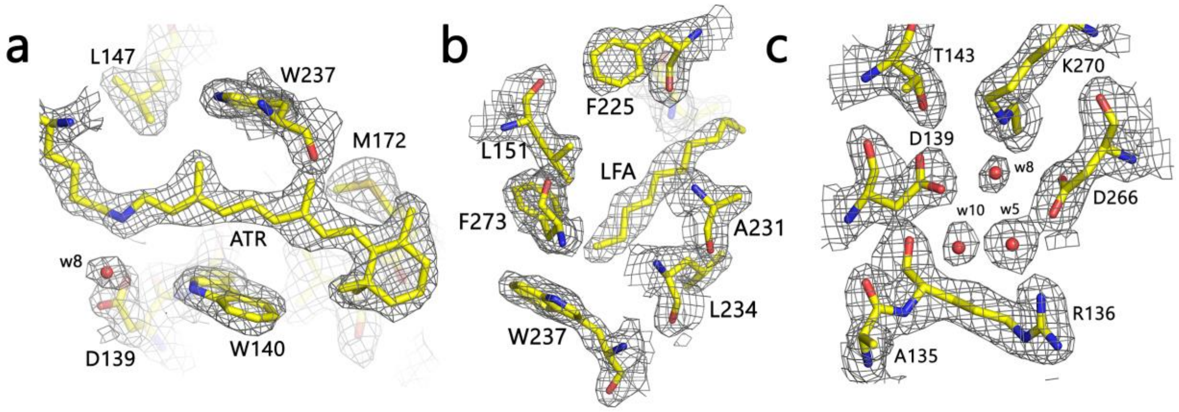

Supplementary Figure 2. Examples of 2Fo-Fc electron density maps. LR electron maps in (a) retinal binding pocket, (b) lipid pocket in intracellular part, and (c) Schiff base regions. Maps are contoured at the 1.5σ level and presented with gray mesh. Water molecules are depicted as red spheres.

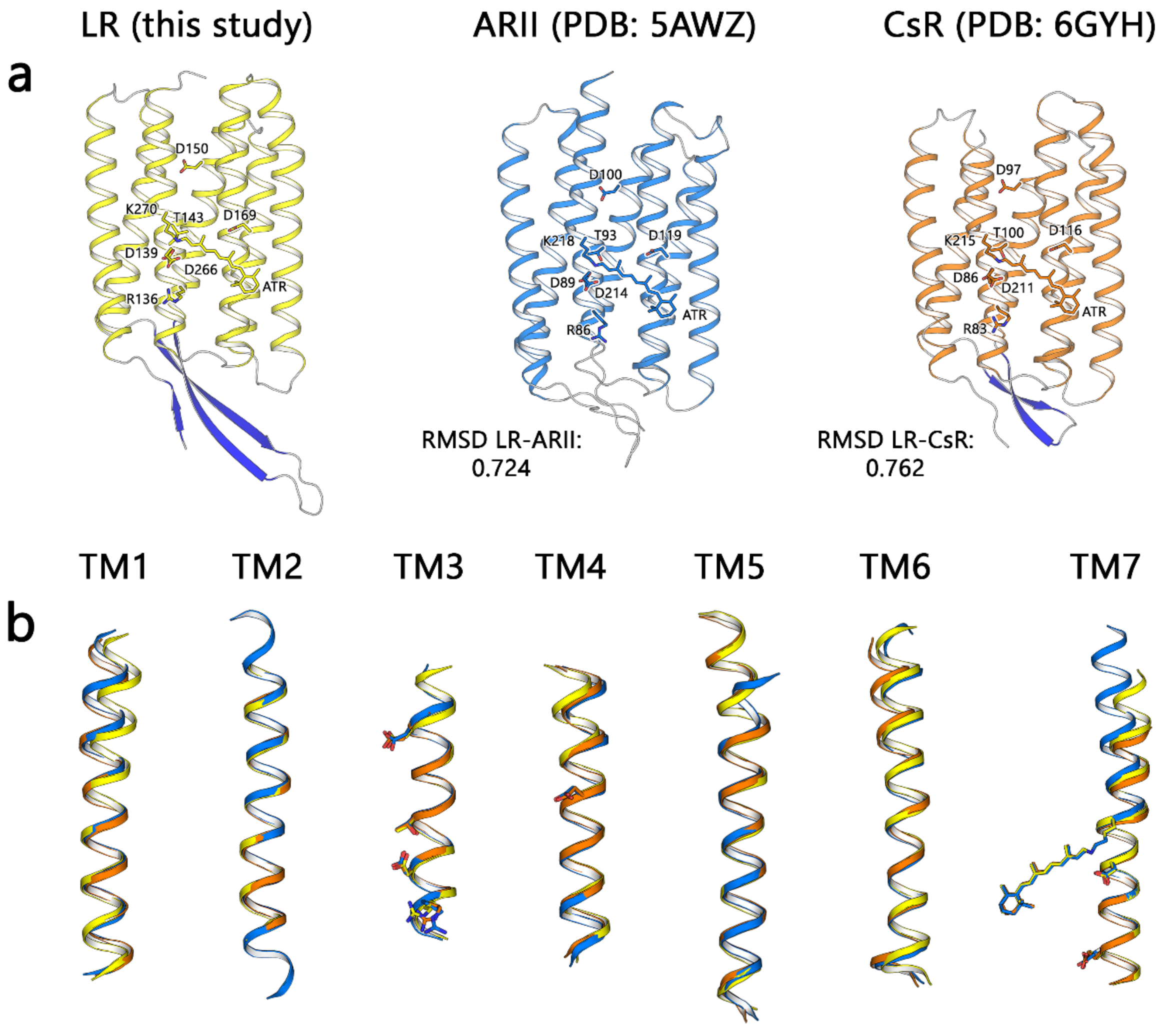

Supplementary Figure 3. Comparison of LR with other eukaryotic proton pumps. (a) High-resolution structures of fungal rhodopsin LR (this study), ARII rhodopsin from marine algae (PDB ID: 5AWZ), and CsR rhodopsin from green algae (PDB ID: 6GYH). Functionally important residues and retinal cofactor are drawn as sticks. Pair RMSD values between proteins are additionally indicated. (b) Individual TM helices are shown after the superimposition of the LR and with ARII and CsR rhodopsins.

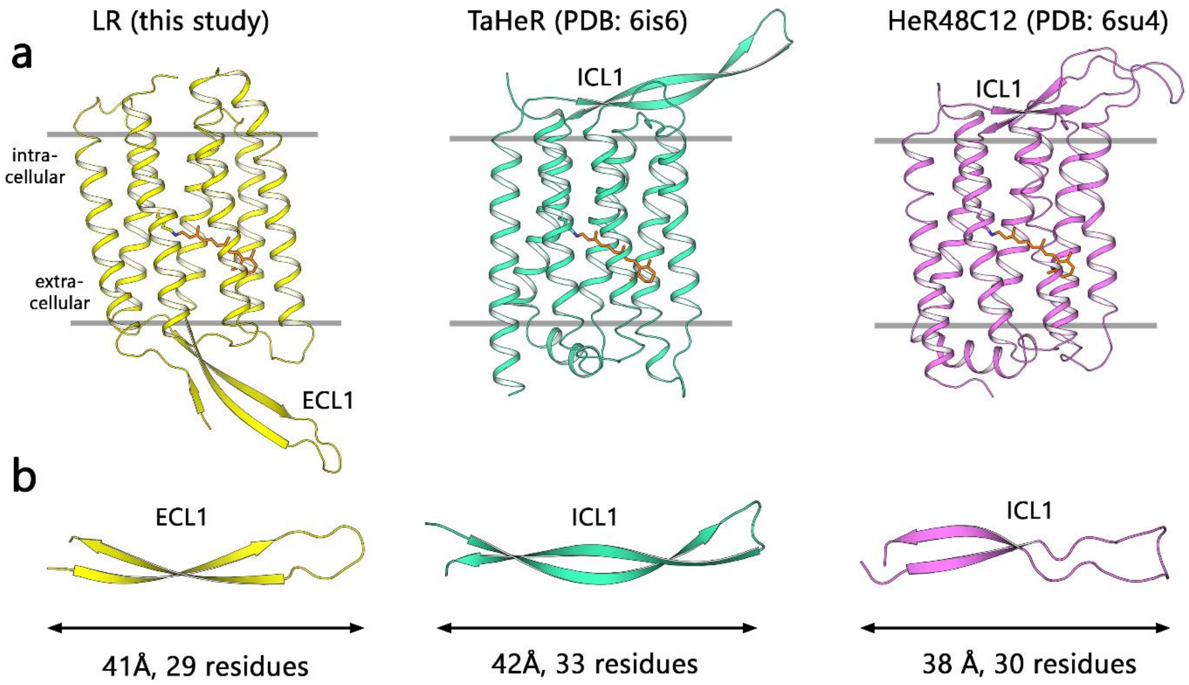

Supplementary Figure 4. Comparison of LR with heliorhodopsins. (a) Crystal structure of LR (left), *Ta*HeR[1] (middle) and HeR-48C12[2] (right) monomers viewed parallel to membrane. (b) Magnified view of elongated beta sheet domains, ECL1 of LR, and ICL1 of *Ta*HeR and HeR48C12.

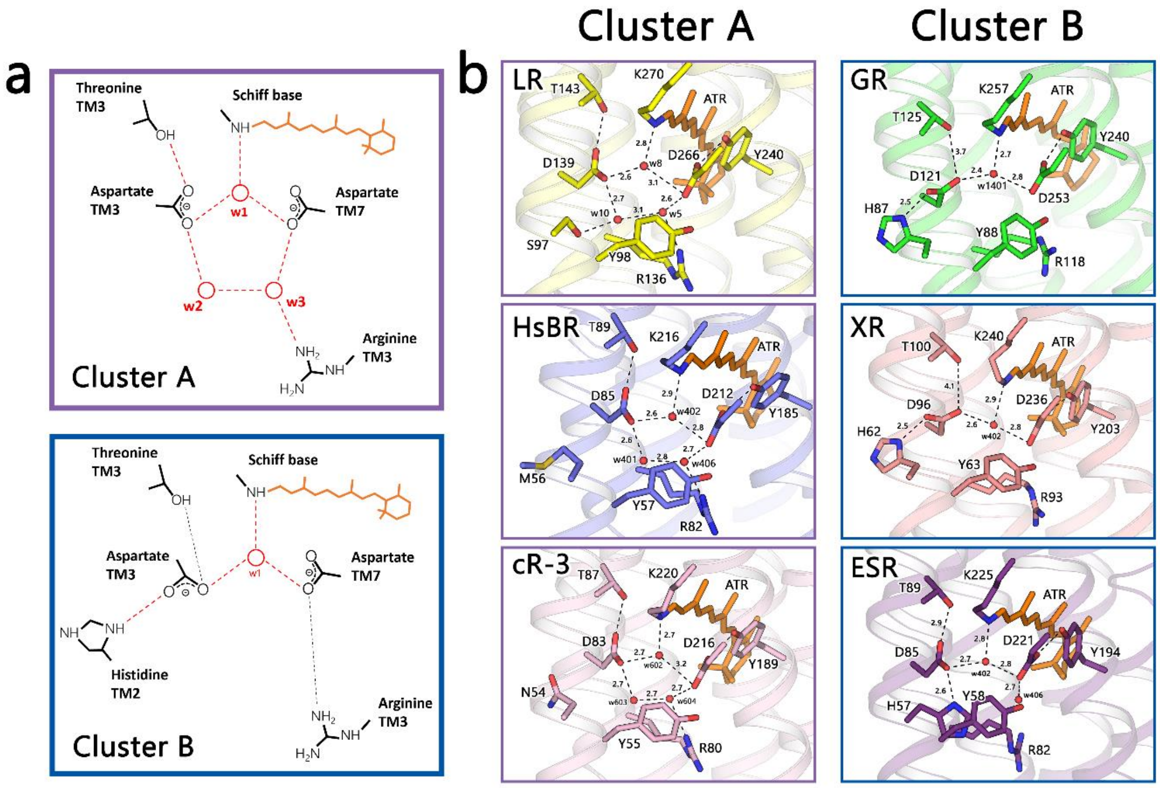

Supplementary Figure 5. Detailed comparison of Schiff base (SB) regions. (a) Schematic representation of Schiff base region configuration in rhodopsins from Cluster A (archaeal and eukaryotic rhodopsins) and Cluster B (bacterial rhodopsins). Sidechains of key residues presented black, water molecules are depicted as red spheres, hydrogen bonds with distance less than 3 Å are shown as red dashed lines. Cluster A rhodopsins have 3 characteristic water molecules near the Schiff base, whereas Cluster B rhodopsins have only one water molecule in SB region. (b) Representative examples of Schiff base regions in rhodopsins from Cluster A (left) and Cluster B (right). Leptosphaeria rhodopsin (LR, this study), halobacterium salinarum rhodopsin (*Hs*BR, PDB ID: 1C3W), cruxrhodopsin-3 (cR-3, PDB ID: 4L35), gloeobacter rhodopsin (GR, PDB ID: 6NWD), xanthrorhodopsin (XR, PDB ID: 3DDL) and exiguobacterium sibiricum rhodopsin (ESR, PDB ID: 4HYJ) were used for the comparison. Key residues were drawn with sticks, water molecules are presented as red spheres and hydrogen bonds are shown as dashed lines.

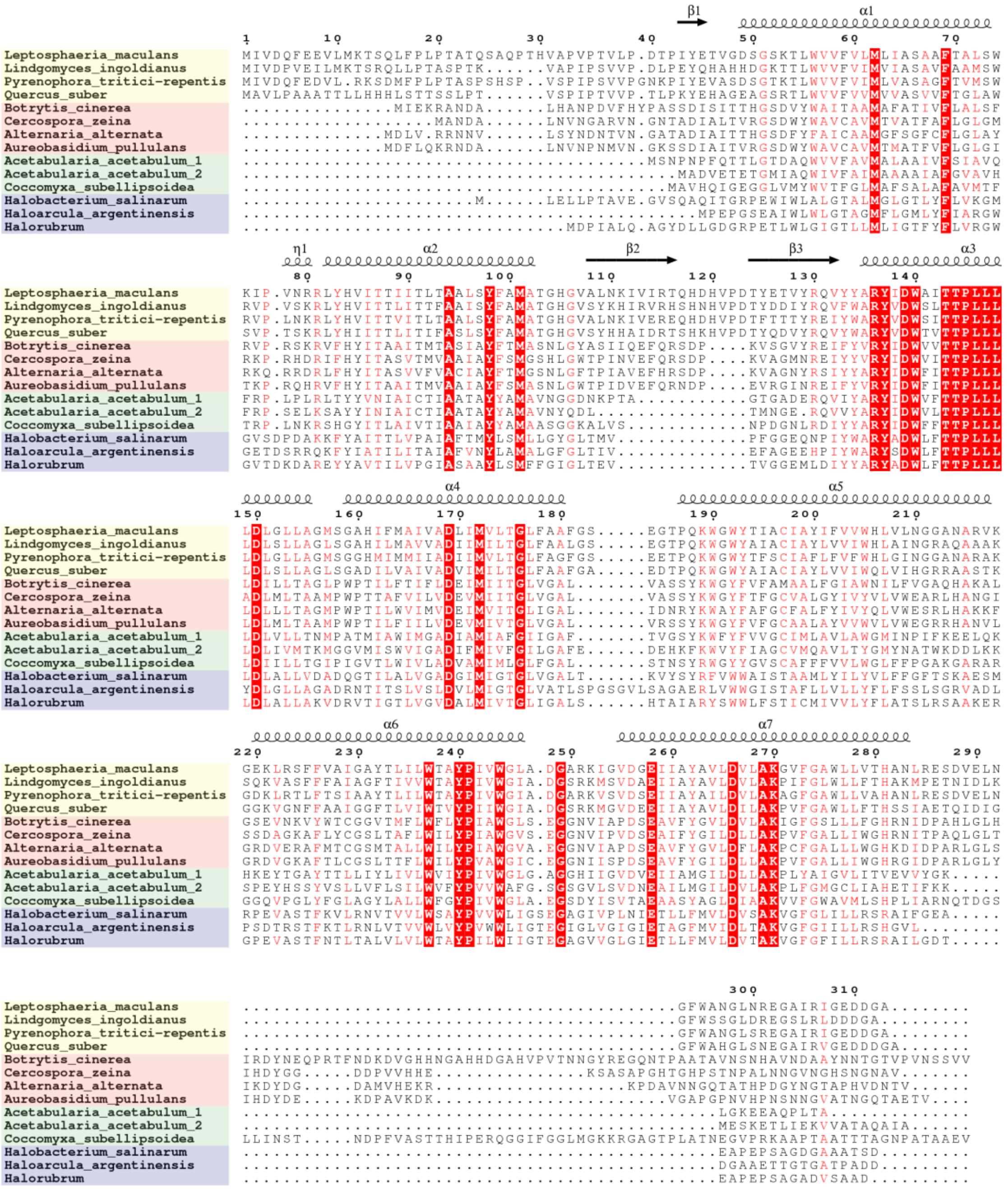

Supplementary Figure 6. Sequence alignment of selected light-driven proton pumps. Sequence alignment was created with MUSCLE and visualized using ESPript 3.0 server[3]. Secondary structure elements for LR structure are shown as coils and arrows. Highly conserved amino acids are highlighted with red color. Following sequences were used for the alignment: Leptosphaeria maculans (LR, AAG01180.1), Lindgomyces ingoldianus (XP_033553403.1), Pyrenophora tritici-repentis (XP_001937307.1), Quercus suber (XP_023878989.1), Botrytis cinereal (XP_001547284.1), Cercospora Zeina (PKR97444.1), Alternaria alternata (OWY42043.1), Aureobasidium pullulans (THY64638.1), Acetabularia acetabulum (Ace-1, AEF12206.1 and Ace-2, AEF12207.1), Coccomyxa subellipsoidea (CsR, EIE22144.1),

Halobacterium salinarum (*Hs*BR, WP_010903069.1), Haloarcula argentinensis (Crux-1, sp|Q57101.1), Halorubrum (Arch-3, WP_092921078.1).

Helix		B	C	C	C	C	C	C	C	C	D	E	F	F	-	G	G	G	G
# in BR		56	82	83	85	86	89	90	93	96	115	125	182	189	194	204	212	215	216
BR	Archaeal pumps	M	R	Y	D	W	T	T	L	D	D	G	W	W	E	E	D	A	K
HwBR/MR		S	R	Y	D	W	T	T	L	D	D	T	W	W	E	E	D	A	K
aR-1		A	R	Y	D	W	T	T	L	D	D	A	W	W	E	E	D	A	K
aR-2		A	R	Y	D	W	T	T	L	D	D	A	W	W	E	E	D	A	K
aR-3		A	R	Y	D	W	T	T	L	D	D	A	W	W	E	E	D	A	K
cR-1		N	R	Y	D	W	T	T	L	D	D	T	W	W	E	E	D	A	K
cR-3		N	R	Y	D	W	T	T	L	D	D	T	W	W	E	E	D	A	K
dR-3		M	R	Y	D	W	T	T	L	D	D	A	W	W	E	E	D	A	K
LR/Mac	Fungal and Algal pumps	S	R	Y	D	W	T	T	L	D	D	A	W	W	D	E	D	A	K
ARII/Ace2		A	R	Y	D	W	T	T	L	D	D	A	W	W	S	E	D	A	K
AR		A	R	Y	D	W	T	T	L	D	D	A	W	W	A	E	D	A	K
CsR		A	R	Y	D	W	T	T	L	D	D	A	W	W	E	E	D	A	K
Ph2		S	R	Y	D	W	T	T	L	D	D	A	W	W	D	E	D	A	K
Ph1		A	R	Y	D	W	T	T	L	D	D	A	W	W	E	E	D	A	K
ESR	Bacterial pumps	H	R	Y	D	W	T	T	L	K	D	E	W	Y	F	R	D	N	K
Med12		H	R	Y	D	W	T	V	Q	E	S	E	W	Y	F	L	D	N	K
HOT75		H	R	Y	D	W	T	V	Q	E	S	E	W	Y	L	L	D	N	K
XR		H	R	Y	D	W	T	V	L	E	A	E	W	Y	A	L	D	A	K
TR		H	R	Y	D	W	T	V	L	E	S	E	W	Y	W	I	D	A	K
gPR		H	R	Y	D	W	T	V	L	E	S	E	W	Y	L	L	D	N	K
MacR		H	R	Y	D	W	T	V	M	E	A	E	W	Y	V	R	D	N	K
PR from O.marina		H	R	Y	D	W	T	V	L	E	S	E	W	Y	I	E	D	A	K
GR		H	R	Y	D	W	T	V	L	E	S	E	W	Y	L	V	D	A	K
GPR1		H	R	Y	D	W	T	V	M	E	S	E	W	Y	P	L	D	N	K
GPR2		H	R	Y	D	W	T	V	L	E	T	E	W	Y	M	L	D	N	K
GPR3		H	R	Y	D	W	T	V	L	E	T	E	W	Y	M	L	D	N	K
BPR		H	R	Y	D	W	T	V	Q	E	S	E	W	Y	F	L	D	N	K
NM-R1		H	R	Y	D	W	T	V	M	E	S	E	W	Y	P	L	D	N	K

Supplementary Figure 7. Conservation of the key amino acids for H^+ pumps. The sequence alignment was performed with MUSCLE. A subset of residues that are conservative and play a role in protein activity was chosen. The numbering scheme is done accordingly with the position in BR (PDB ID: 1C3W). Negatively charged residues are shown in reds, positively charged in blues, aromatic in yellow and orange, polar in greens, non-polar in greys.

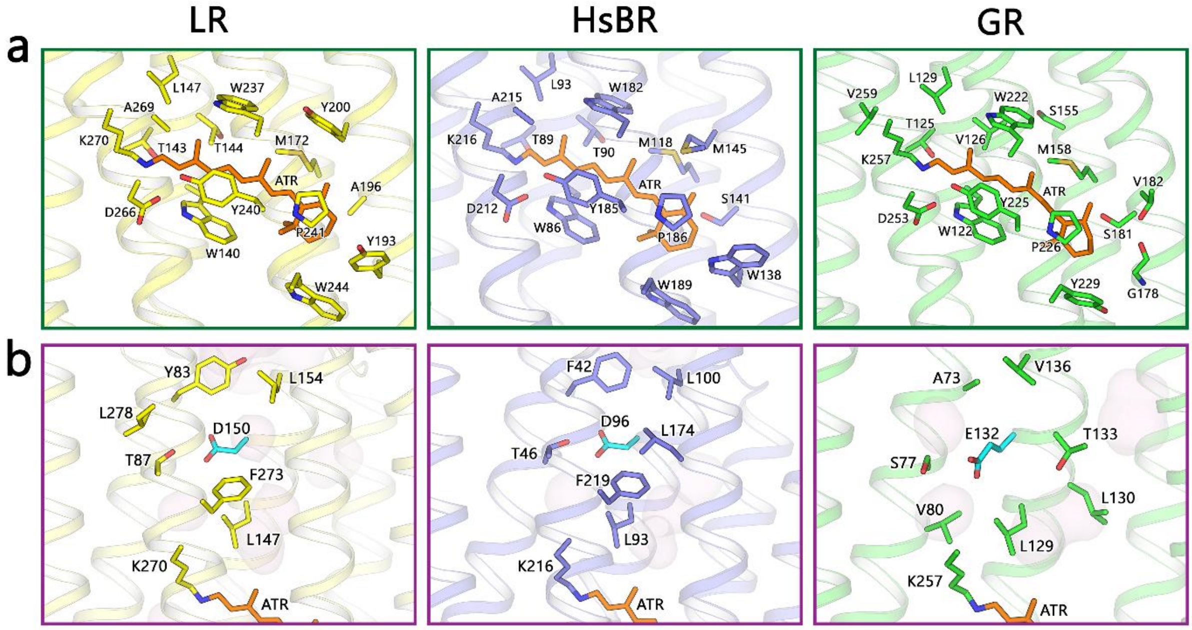

Supplementary Figure 8. Proton donor region of LR. Magnified view of (a) retinal pocket and (b) proton donor regions of LR (left), *Hs*BR (center) and GR (right). Important residues are shown with sticks, retinal chromophore and proton donor residues are colored orange and cyan respectively.

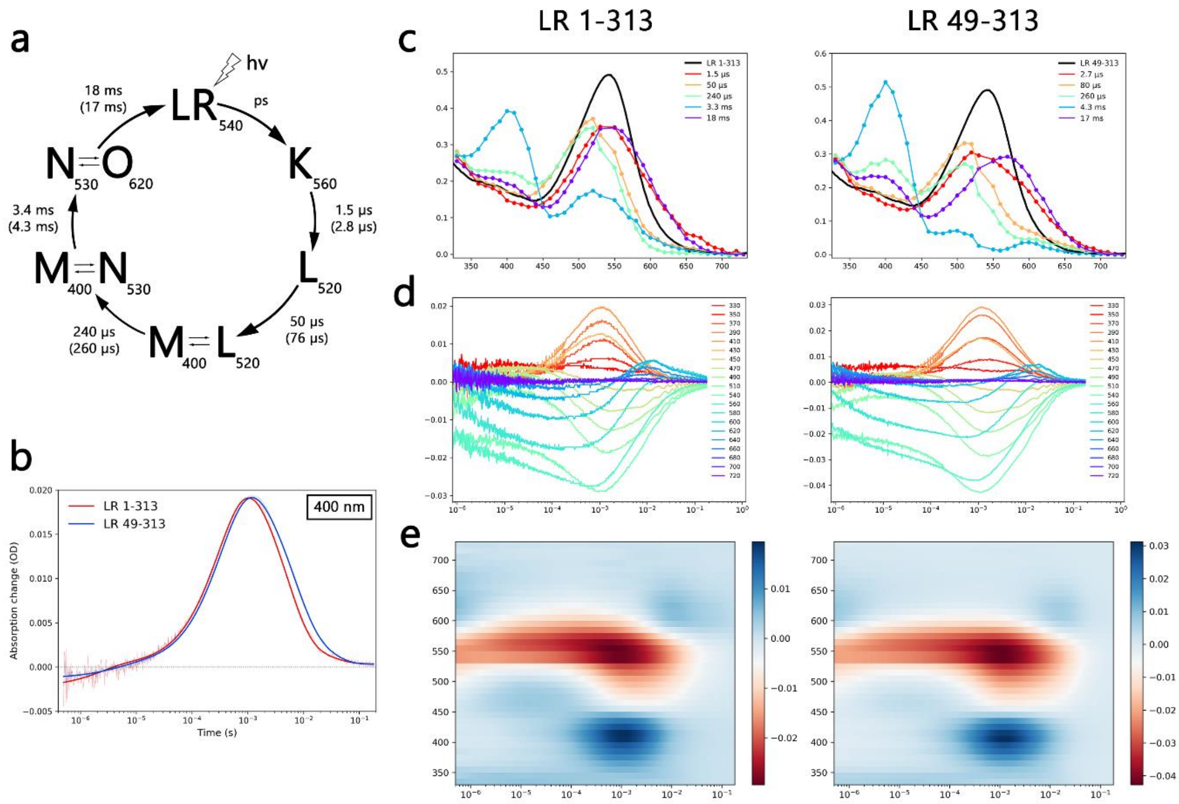

Supplementary Figure 9. Comparison of photocycle kinetics of LR constructs. (a) Schematics representation of LR photocycle kinetics, lifetimes of LR 1-313, and LR 49-313 are shown without and with brackets respectively. (b) Time traces of absorption changes of LR constructs at 400 nm wavelength. (c) Absorption spectra of LR 1-313 (left) and LR 49-313 (right) photocycle intermediates. The ground spectra are drawn in black. (d) Time traces of absorption changes of LR constructs. (e) Contour plots of transient absorption changes of LR constructs. Both samples were measured n = 2 times.

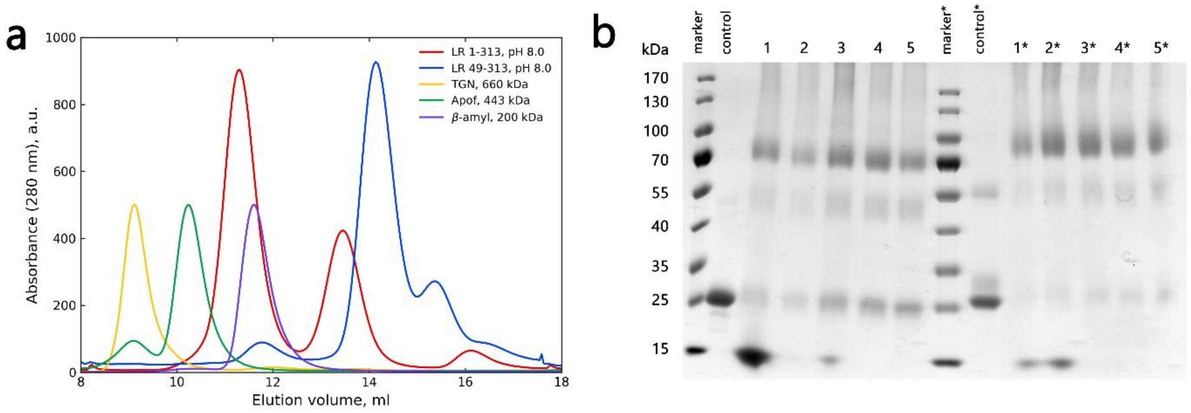

Supplementary Figure 10. Full cross-linking analysis of LR protein. (a) Glutaraldehyde crosslinking of LR 1-313 and LR 49-313 was carried out as described previously[4]. Gradient SDS–PAGE 18.5-8%. Lanes 1-5 and 1*-5*: Influence of glutaraldehyde vapor on the protein solution at different time intervals: 30, 60, 90, 120, and 180 min in case of LR 49-313 and LR 1-313 correspondingly. After 30 min incubation bands corresponding to dimer and trimer can be observed. LR samples not treated with glutaraldehyde were used as a control. (b) Size-exclusion chromatography profiles of LR 1-313 and LR 49-313 protein used for crystallization trials. SEC profiles for calibration proteins (apoferritin, beta-amylase, phosphorylase, and bovine serum albumin) are additionally presented. Elution volumes of calibration proteins were used for the calculation of the expected size of DDM micelles with LR protein.

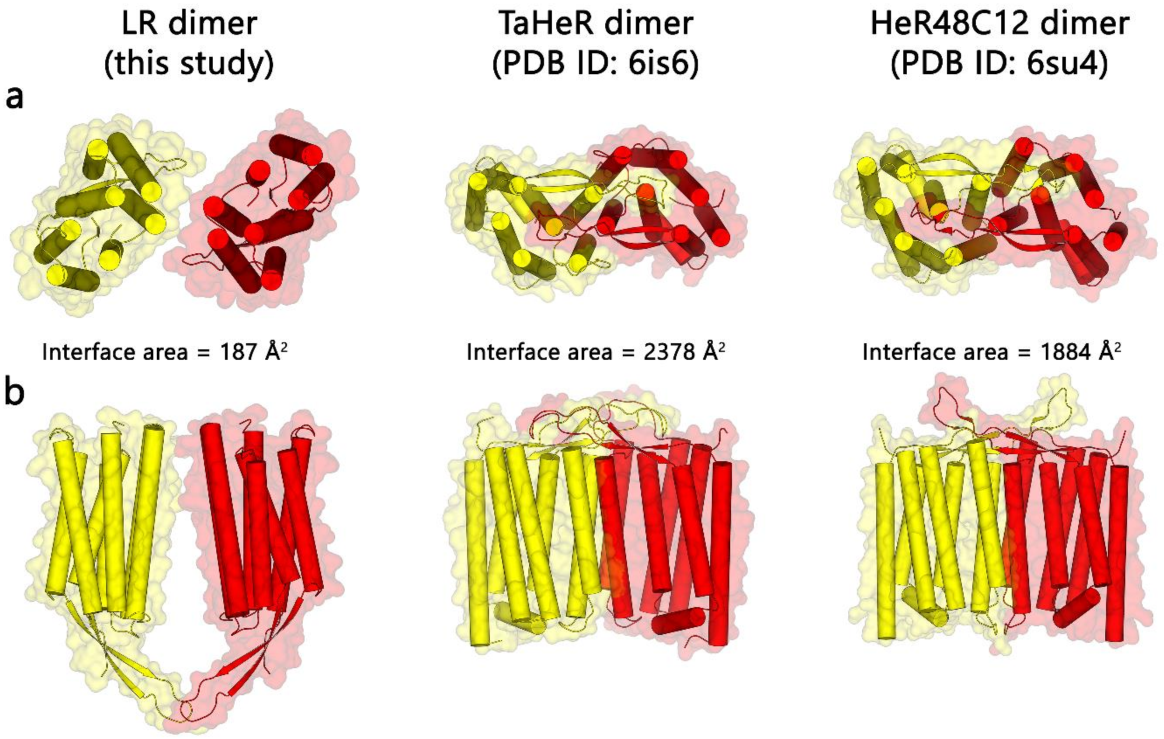

Supplementary Figure 11. Comparison of dimer configuration of LR and HeRs. (a) Crystal structure of dimers of LR (left), *Ta*HeR[1] (middle), and HeR48C12[2] (right) monomers viewed from (a) intracellular side and (b) parallel to the membrane. Individual monomers are colored red and yellow, interface surface area is indicated for all proteins.

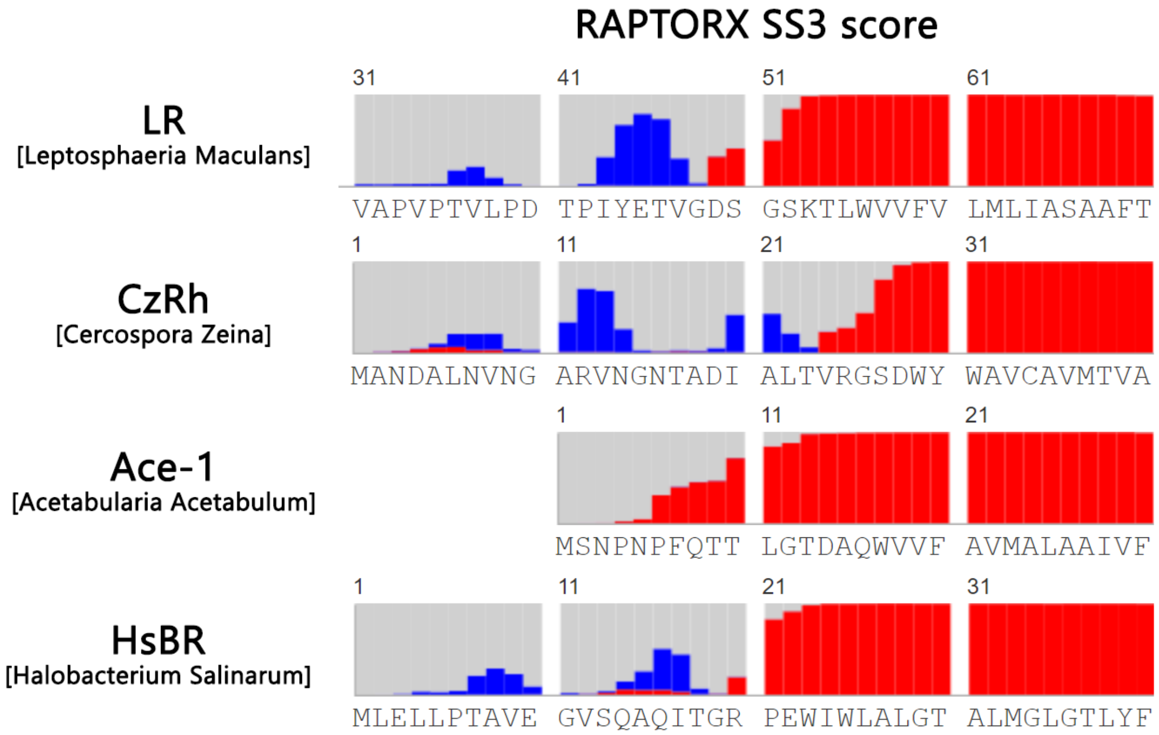

Supplementary Figure 12. Secondary structure prediction for selected proton pumps. (a) Predicted secondary structure calculated using RaptorX web server[5]. Residues 43-46 of LR are predicted to form a beta-strand, which was observed in the crystal structure. No beta strands at N-terminus were predicted for other rhodopsins, besides a possible beta-strand for *Hs*BR, which is not supported by structural data.

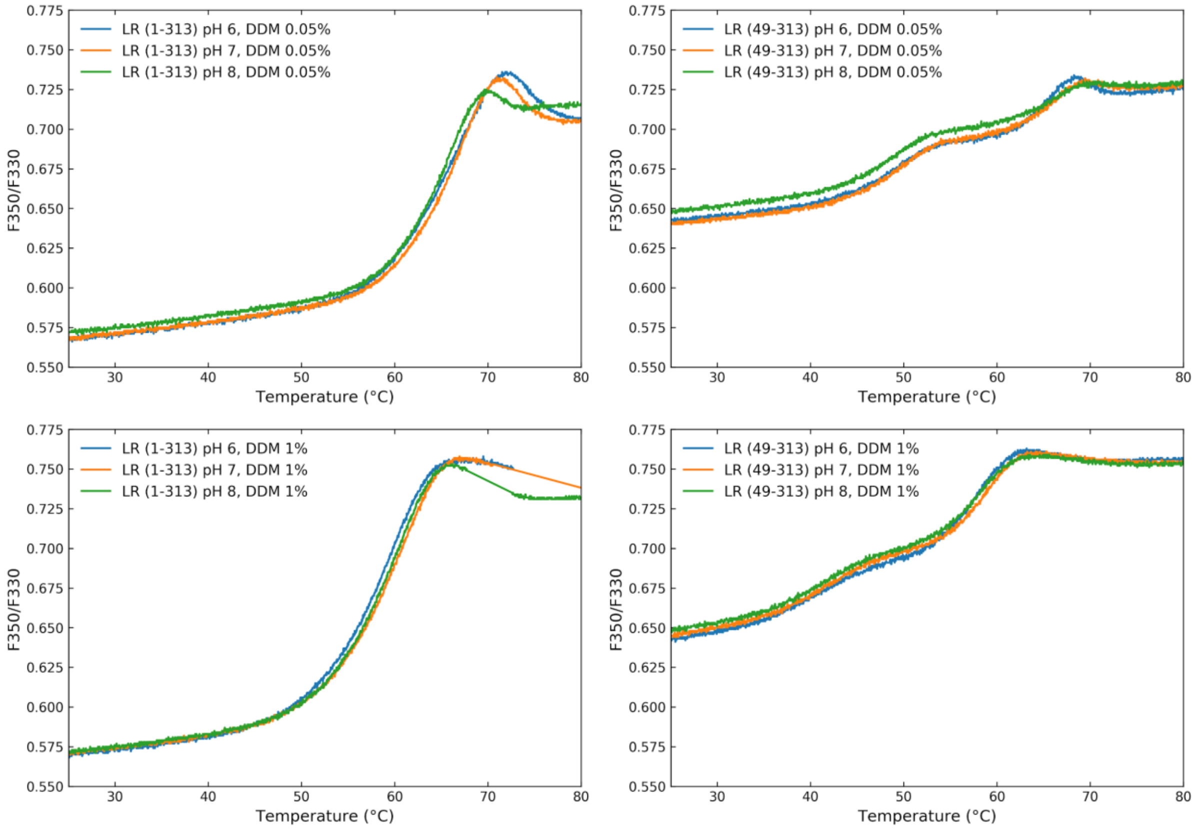

Supplementary Figure 13. NanoDSF spectra of LR. Differential fluorimetry spectra measurements are presented for LR 1-313 and LR 49-313 under different pH (pH 6.0, pH 7.0 and pH 8.0) and detergent (0.05% DDM, 1 % DDM) conditions. Corresponding spectra for the protein in different pH are colored blue, orange and green for pH 6.0, pH 7.0 and pH 8.0 respectively. For all the samples, n = 1.

Supplementary Table 1. List of plasmids used in this study.

pBKT plasmid (gene position described as xxx)	TCCCTATCAGTGATAGAGATCCAGTCGCAGCCTGACCGCATCACACATCAAGGCGTTACAGCCTCTCCGTCTTTGCTGCACCC<br>AAGTGTATTCGTGTGACCGCGTTGAGGGATCTAATGGATTCGGAACTTTGGTCGTTTGGTCTCTCATGCTGCCCAATCCCATG<br>CGCTTTTCTCCGTGCCTCTCTGTCTCCCCTTCCGTTCTATTTGGACAACGATGTGTACTGTAATGCGTGCGCCATCTGACAACG<br>AATAGATCAGCAGCATTCGCACACTTGCACATACCCAGTGAAGCTTTTGTGTCTGTCGTATTGACAACACCGACTGCAACAA<br>GGTGTAGATAGAAGTTGGCCTTCTCGCTCGCTCGCACGCTCTTCACGCTCCTGCTTTCCTTGCTGTGCCTTGCCACCAGATCTG<br>CCxxxxxxxxxxxxCACCACCATCACCACCACTAGGCGGCCGCCCTCCTCCTCCTTTCTTGTTCCTTTCACGTCGCCTTCTCGGTTG<br>TAGCTGGCAGACGACGAGTCTTACTTTTACGTGTACTTCTCTATAGATGATGTATGATCTCTCTGCATGCGTGTTCGTGCATG<br>TGTCCGTGTGTTGTGTACGCGTGCGTCTCGCCTCAGCTCTCCGCGTGAAAGGGTTTGACTGCCCATGATGCGTGTGTATATCC<br>ACGCGCAGGCACGCACACACACACACACACACACACACACAGGCACACACAGGCACACAAACGCATCTCAGGCCGAGCCG<br>CATACGTCTCTCGCACGGTCTCGTTTATTTGATCATGTAGTTGAGTATTAAATTGGGAAGACAAAAACATAATAGCACGAAG<br>AGTCGGGCACGAAAAGCCCGATCTCTCTCTCTCTCTCTCGTGCGCGCAGGGGCGTGTGGGTGCACGACGACGAGGACGGAG<br>GGGGGAAGGGAGGCATAAACGGATCGATCCCCCATCGCACATGCGGGTACGAGCATTATCTGCTGTGTCTGGTCCTTTATCA<br>TATCGCTACCCGCCCGCCCCCCGCCCCCCTCCCCCCCCCCCCCCCGCGGCTCCTGTCACTGTCGTTCCTGCGACTCCCCACA<br>CACCCGCGCAACGCTCATGTCACGAAGAGAAAAGTAGAAGGCGTGGCGATGCGTTGCGCGGCCCCCTGTCCGCTGGCATGC<br>AGACGTCGAGCCGTGGAACGGATGGTAGCGAGAGACAACGCGGCGATGCAGGAAAGGAGATTTACTGCAGGACGTCTACA<br>CACGCGCGCACACCACTGCGAATGGCACCGTGCTGAGGAAAATTGGGGGGGAGGGCGCACGGGGGCGGGGCAGGAAACGG<br>TTGGATCAGCAGCACTCTCAACTTGTGTCCGTATTGCGAAGGAGGGAGAGGAGCCTGCCCGTATTATGGGCGCTGAAACGTC<br>GGAACCACCTATGAATCCCACTTCTGCGTCGCGGAGGCGTACTGCTTCCGACCACCTCCGCAGCTCACGCGCGTCGAGCTCC<br>CTCCCTTTCCCCTTCTTTCCGTTGTGTGTGTGTGTGTGTGTGTGTCTTGTCACGATGCATGGCCATCTCTCCTCCAACCCTCCAC<br>GCCTTCCTCTCCCCCGCCCATCAAACGCGCTACGGCCACAACCATTTCTTCAAGTATCACATCCACCACCACTCTTACCCACC<br>TACCCTCTTCGACTCTGACACAGCCACTGACGCCCTCTCCGCCTCTCTCGCTCGCTGCACATTGACTCTCCACAGCCCCCCCTC<br>CCTCGCACAGCCAGCCCCACCCACCCACCCACCCACCCACCGCTCTACACACCTCCACAGCAGCCGGATCCACCGCATGGCC<br>AAGTTGACCAGTGCCGTTCCGGTGCTCACCGCGCGCGACGTCGCCGGAGCGGTCGAGTTCTGGACCGACCGGCTCGGGTTCT<br>CCCGGGACTTCGTGGAGGACGACTTCGCCGGTGTGGTCCGGGACAACGTGACCCTGTTCATCAGCGCGGTCCAGGACCAGGT<br>GGTGCCGGACAACACCCTGGCCTGGGTGTGGGTGCGCGGCCTGGACGAGCTGTACGCCGAGTGGTCGGAGGTCGTGTCCAC<br>GAACTTCCGGGACGCCTCCGGGCCGGCCATGACCGAGATCGGCGAGCAGCCGTGGGGGCGGGAGTTCGCCCTGCGCGACCC<br>GGCCGGCAACTGCGTGCACTTCGTGGCCGAGGAGCAGGACGTCGAGTCCGGAATGGTGAGCAAGGGCGAGGAGGATAACA<br>TGGCCATCATCAAGGAGTTCATGCGCTTCAAGGTGCACATGGAGGGCTCCGTGAACGGCCACGAGTTCGAGATCGAGGGCG<br>AGGGCGAGGGCCGCCCCTACGAGGGCACCCAGACCGCCAAGCTGAAGGTGACCAAGGGTGGCCCCCTGCCCTTCGCCTGGG<br>ACATCCTGTCCCCTCAGTTCATGTACGGCTCCAAGGCCTACGTGAAGCACCCCGCCGACATCCCCGACTACTTGAAGCTGTCC<br>TTCCCCGAGGGCTTCAAGTGGGAGCGCGTGATGAACTTCGAGGACGGCGGCGTGGTGACCGTGACCCAGGACTCCTCCCTGC<br>AGGACGGCGAGTTCATCTACAAGGTGAAGCTGCGCGGCACCAACTTCCCCTCCGACGGCCCCGTAATGCAGAAGAAGACTA<br>TGGGCTGGGAGGCCTCCTCCGAGCGGATGTACCCCGAGGACGGCGCCCTGAAGGGCGAGATCAAGCAGAGGCTGAAGCTGA<br>AGGACGGCGGCCACTACGACGCTGAGGTCAAGACCACCTACAAGGCCAAGAAGCCCGTGCAGCTGCCCGGCGCCTACAACG<br>TCAACATCAAGTTGGACATCACCTCCCACAACGAGGACTACACCATCGTGGAACAGTACGAACGCGCCGAGGGCCGCCACT<br>CCACCGGCGGCATGGACGAGCTGTACAAGTAGACTAGTTCTAGTTCTAGGGGCCGAATTAATTCAGATCCTCGTGTGAGCGT<br>TCGCGGAATCGGTCGCTCGTGTTTATGCCCGTCTTGGTGTTGTGCTCGCAAGGCGGTGCAGCAGGATACCGTCGCCCTCCTCT<br>CTCCTTGCTTCTCTGTTCTTCAATTCGCGATCTCACAGAGGCCGGCTGTGCACGCCCTTCCTCACCCTCCTTTCCCACCTCTC<br>GGCCACCGGTCGGCTCCGTTCCGTCTGCCGTCGAGAAGGGACGGGCATGTGCAGCTCCTCCCTTTCTCTCGCGCGCGCATCTT<br>CTCTTGCTTGTGGCACTCACGCTCATGCGTCAAGGCGGCCCCACGCCGAGCCCCTGCGCTCCCTTCCCTCTTGCGCATCCGTA<br>GCGCGGATGCCGTCGATGCGCAAGGCCGGCATGAAGGAGCGCGTGCCCTCAAGAGGGCACACTATCATGCCCTACGTGGGC<br>CACGCAGCGATGAGGCCGGCTTCGGCGGAGATGCGTCACGCACGTGCCAGATGATGCGCTACGCCTTCCTTGACTTGCGCCC<br>CCCTCTCTTCCTCCGTCTCTCACTCTCTCTCTCTCACACACACACACACACACACACACACACAAAGCTCCGGTTCGTCCGGG<br>CCCTGCAGGCATGCCGACGTTCGGTGACCTGAACCACCTCGTCGCCGCTGTGATGTCTGGCGTGACCTGCTGCCTGCGCTTCC<br>CTGGCCAGCTGAACTCTGACCTGCGCAAGCTTCTCCTGCTCAACGACGGCGACGGCGCTGACGAGGTGCAGAGTGGCACAG<br>AAGCCGCAGCGGCGTACAGCGACGAGGAGGGCGGGAAGTCGCCGAGTGGTCCCCTGGCGAACGACTCCCTGTTCATGTCCG<br>CGTGGGACCGCCGTCGCAACCTCACGAGCCGCCCGCTGCGCATCACAACCATATTCGGCCCCACCTGCGATTCGATGGACTG<br>CATTCTGAAGAAGCAGCCCTTCCCGGAGATGAAGCTCGGCGACTGGCTTCTAGTGCCAGACATGGGCAGCTACACCACCGCT<br>GCCGCGGGAGTCTTCAACGGTTTCGCGACACGCCGCCTTGAGTTTGTGAGCTCTGTTGACTTGTCTGCGAGGTCGAGGGCTGT<br>GCATGCGCATGAGGGCAACACTTTTGAGTTCGTGAGCGAGTGAAAACGGGGCTGAGAGGATACAAGGTGCTGTAGGATGAG<br>ATCCTCCGCCCACCAGAGCATGGGCTATCTCTTGTGTCTGTTCTGAGCCTGCCTCTCTCTCTCTCTCTTGCCTCCCCCTCCCCC<br>CTCCCCACACACACCCCACACGCACGCTCTGCGTCACCCTCTCCTCCCTCAGTAACGGGAGTAGACATGCAGAAGAAGTATA<br>CCACTAATACGTATGCGTTCCTGCAGGCATCCGAGATGGACACCAGAGCAAGCGCGGGTGTGCGCGAGTGGCCAGGAAAGG<br>GGAAGAGAGAGAAAGAGAGAGAGACGAAGGAGCGCGTCCCGTCGTTGTGCGTCTTCTGTGGTGGCGCATTTAAATTTAGAG<br>CTTGACGGGGAAAGCCGGCGAACGTGGCGAGAAAGGAAGGGAAGAAAGCGAAAGGAGCGGGCGCTAGGGCGCTGGCAAG<br>TGTAGCGGTCACGCTGCGCGTAACCACCACACCCGCCGCGCTTAATGCGCCGCTACAGGGCGCGTCAGGTGGCACTTTTCGG<br>GGAAATGTGCGCGGAACCCCTATTTGTTTATTTTTCTAAATACATTCAAATATGTATCCGCTCATGAGACAATAACCCTGATA<br>AATGCTTCAATAATATTGAAAAAGGAAGAGTATGAGTATTCAACATTTCCGTGTCGCCCTTATTCCCTTTTTTGCGGCATTTT<br>GCCTTCCTGTTTTTGCTCACCCAGAAACGCTGGTGAAAGTAAAAGATGCTGAAGATCAGTTGGGTGCACGAGTGGGTTACAT<br>CGAACTGGATCTCAACAGCGGTAAGATCCTTGAGAGTTTTCGCCCCGAAGAACGTTTTCCAATGATGAGCACTTTTAAAGTT<br>CTGCTATGTGGCGCGGTATTATCCCGTATTGACGCCGGGCAAGAGCAACTCGGTCGCCGCATACACTATTCTCAGAATGACT<br>TGGTTGAGTACTCACCAGTCACAGAAAAGCATCTTACGGATGGCATGACAGTAAGAGAATTATGCAGTGCTGCCATAACCAT<br>GAGTGATAACACTGCGGCCAACTTACTTCTGACAACGATCGGAGGACCGAAGGAGCTAACCGCTTTTTTGCACAACATGGGG<br>GATCATGTAACTCGCCTTGATCGTTGGGAACCGGAGCTGAATGAAGCCATACCAAACGACGAGCGTGACACCACGATGCCT<br>GTAGCAATGGCAACAACGTTGCGCAAACTATTAACTGGCGAACTACTTACTCTAGCTTCCCGGCAACAATTAATAGACTGGA<br>TGGAGGCGGATAAAGTTGCAGGACCACTTCTGCGCTCGGCCCTTCCGGCTGGCTGGTTTATTGCTGATAAATCTGGAGCCGG<br>TGAGCGTGGGTCTCGCGGTATCATTGCAGCACTGGGGCCAGATGGTAAGCCCTCCCGTATCGTAGTTATCTACACGACGGGG<br>AGTCAGGCAACTATGGATGAACGAAATAGACAGATCGCTGAGATAGGTGCCTCACTGATTAAGCATTGGTAACTGTCAGAC<br>CAAGTTTACTCATATATACTTTAGATTGATTTAAAACTTCATTTTTAATTTAAAAGGATCTAGGTGAAGATCCTTTTTGATAAT<br>CTCATGACCAAAATCCCTTAACGTGAGTTTTCGTTCCACTGAGCGTCAGACCCCGTAGAAAAGATCAAAGGATCTTCTTGAG

	ATCCTTTTTTTCTGCGCATAATCTGCTGCTTGCAAACAAAAAAACCACCGCTACCAGCGGTGGTTTGTTTGCCGGATCAAGAG<br>CTACCAACTCTTTTTCCAAGGTAACTGGCTTCAGCAGAGCGCAGATACCAAATACTGTTCTTCTAGTGTAGCCGTAGTTAGGC<br>CACCACTTCAAGAACTCTGTAGCACCGCCTACATACCTCGCTCTGCTAATCCTGTTACCAGTGGCTGCTGCCAGTGGCGATAA<br>GTCGTGTCTTACCGGGTTGGACTCAAGACGATAGTTACCGGATAAGGCGCAGCGGTCGGGCTGAACGGGGGGTTCGTGCAC<br>ACAGCCCAGCTTGGAGCGAACGACCTACACCGAACTGAGATACCTACAGCGTGAGCTATGAGAAAGCGCCACGCTTCCCGA<br>AGGGAGAAAGGCGGACAGGTATCCGGTAAGCGGCAGGGTCGGAACAGGAGAGCGCACGAGGGAGCTTCCAGGGGGAAAC<br>GCCTGGTATCTTTATAGTCCTGTCGGGTTTCGCCACCTCTGACTTGAGCGTCGATTTTTGTGATGCTCGTCAGGGGGGCGGAG<br>CCTATGGAAAAACGCCAGCAACGCGGCCTTTTTACGGTTCCTGGCCTTTTGCTGGCCTTATTTAAATGAATACTTACAAGGTA<br>CTCCTGCACCCGGCACAAGCGTATGTAATTGCTAGCACGCCGGTGCCGACCCAACCTCCAACTCCCGTGCTCACGCGCGCAC<br>ACACGCACGCACAAGCATCATCATCATCATCATCATGGTCATCCCTCCCTTATCTGGCGAAGGAGGCACTGTGACGCAAGGT<br>TTTCTGTGTCTCTCCTACCCCTCTACTGATTCCTTCTTTCTCTCTTCCTCTTCTCGCACTGCACCCCCCTCCCCCCCCCCACACA<br>CACACACTTGCTGTCATGGCTGTGCGCATTCGCTCACTCTTGTCGTGTCCCGTTTCCCTCATGGCGTCGTCGTCTTCCCCTGCG<br>CAACCACCCACGCTGCACACGCACGCTCCACAGCGCGCTTCGCCTTCTTCGCTCTCTCCCTAGCCTCCTCTCTGTCACCCTCC<br>ACTGCCGCCGCGGTTGGCCGCTCTGCATCCTTTTCCCGCAGCCTTTCCTTTTTTTTTTCACTGTCAGTCTGGCTCATACCCGTG<br>GAGCTGTGTCTCCCCGAGCTTCCCATACACCCACCCCCCACGACCTTTCTCAGCTTCCTTTCTCAGTCGCTTGAGCACGCAGT<br>GCATTCACTTCTTCGTCCCTCAGTTCGCCTCGCTCTCGCTCTCTCTCGCGCGCGCTGCCGTACCCGCCCCTCCTCGTACTCCCC<br>TCCTTCCTCATCCCCACCCCCCCCGTGTGCGCCGGCTCCTCTCTGAATTCTAATACGACTCACTATAGGGAGATCG

Supplementary Table 2. List of protein genes used in this study.

LR_1-313_His8 codon-optimized for *LEXSY* expression	ATGATCGTGGACCAGTTCGAGGAGGTGCTGATGAAGACGAGCCAGCTGTTTCCGCTGCCGACAGCAACACAGAGCGCACAG<br>CCAACACATGTGGCGCCAGTGCCAACAGTGCTGCCGGACACACCAATCTACGAGACAGTGGGCGACAGCGGCAGCAAGACA<br>CTGTGGGTCGTGTTCGTGCTGATGCTGATTGCGAGCGCGGCGTTTACAGCGCTGAGCTGGAAGATTCCAGTGAACCGCCGTC<br>TGTACCACGTGATCACCACCATCATCACGCTGACAGCGGCCCTGAGCTACTTCGCAATGGCAACAGGCCATGGCGTGGCGCT<br>GAACAAGATCGTGATTCGCACACAGCACGACCACGTGCCAGACACGTACGAGACTGTGTACCGCCAGGTGTACTACGCGCG<br>CTACATCGACTGGGCGATTACGACACCACTGCTGCTGCTGGATCTGGGCCTTCTTGCAGGCATGAGCGGCGCACACATCTTC<br>ATGGCGATTGTGGCGGACCTGATCATGGTGCTGACTGGCCTGTTTGCGGCGTTTGGCTCTGAGGGCACACCACAGAAGTGGG<br>GCTGGTACACGATCGCCTGCATTGCCTACATCTTCGTCGTGTGGCACCTGGTGCTGAACGGCGGTGCAAACGCACGTGTGAA<br>GGGCGAGAAGCTGCGCTCTTTCTTTGTGGCGATTGGCGCGTACACGCTGATCCTGTGGACAGCGTACCCAATCGTGTGGGGT<br>CTTGCAGACGGCGCACGCAAGATCGGTGTCGACGGCGAGATTATTGCGTACGCGGTGCTGGACGTGCTGGCGAAGGGCGTG<br>TTCGGTGCATGGCTGCTTGTGACACATGCGAACCTTCGCGAGAGCGACGTCGAGCTGAACGGTTTTTGGGCGAACGGCCTTA<br>ACCGCGAGGGCGCAATTCGTATCGGCGAGGACGATGGTGCAGGTACCcatcaccatCACCACCATCACCACCACTAG
LR_49-313_His8 codon-optimized for *LEXSY* expression	ATGGACAGCGGCAGCAAGACACTGTGGGTCGTGTTCGTGCTGATGCTGATTGCGAGCGCGGCGTTTACAGCGCTGAGCTGGA<br>AGATTCCAGTGAACCGCCGTCTGTACCACGTGATCACCACCATCATCACGCTGACAGCGGCCCTGAGCTACTTCGCAATGGC<br>AACAGGCCATGGCGTGGCGCTGAACAAGATCGTGATTCGCACACAGCACGACCACGTGCCAGACACGTACGAGACTGTGTA<br>CCGCCAGGTGTACTACGCGCGCTACATCGACTGGGCGATTACGACACCACTGCTGCTGCTGGATCTGGGCCTTCTTGCAGGC<br>ATGAGCGGCGCACACATCTTCATGGCGATTGTGGCGGACCTGATCATGGTGCTGACTGGCCTGTTTGCGGCGTTTGGCTCTG<br>AGGGCACACCACAGAAGTGGGGCTGGTACACGATCGCCTGCATTGCCTACATCTTCGTCGTGTGGCACCTGGTGCTGAACGG<br>CGGTGCAAACGCACGTGTGAAGGGCGAGAAGCTGCGCTCTTTCTTTGTGGCGATTGGCGCGTACACGCTGATCCTGTGGACA<br>GCGTACCCAATCGTGTGGGGTCTTGCAGACGGCGCACGCAAGATCGGTGTCGACGGCGAGATTATTGCGTACGCGGTGCTGG<br>ACGTGCTGGCGAAGGGCGTGTTCGGTGCATGGCTGCTTGTGACACATGCGAACCTTCGCGAGAGCGACGTCGAGCTGAACG<br>GTTTTTGGGCGAACGGCCTTAACCGCGAGGGCGCAATTCGTATCGGCGAGGACGATGGTGCAGGTACCcatcaccatCACCACCA<br>TCACCACCACTAG

Supplementary Table 3. List of primers used in this study.

FrwBglIILoLR1	tgaaAGATCTGCCATGATCGTGGACCAGTTCGAGGAGGTG
RevKpnILoLR313	tagattGGTACCTGCACCATCGTCCTCGCCGATACG
FrwBglIILoLR49	tgaaAGATCTGCCATGGACAGCGGCAGCAAGACACTGTGG

Supplementary References

1. Shihoya, W. *et al.* Crystal structure of heliorhodopsin. *Nature* **574**, 132–136 (2019).

2. Kovalev, K. *et al.* High-resolution structural insights into the heliorhodopsin family. *Proc. Natl. Acad. Sci.* 201915888 (2020) doi:10.1073/pnas.1915888117.

3. Robert, X. & Gouet, P. Deciphering key features in protein structures with the new ENDscript server. *Nucleic Acids Res.* (2014) doi:10.1093/nar/gku316.

4. Fadouloglou, V. E., Kokkinidis, M. & Glykos, N. M. Determination of protein oligomerization state: Two approaches based on glutaraldehyde crosslinking. *Anal. Biochem.* (2008) doi:10.1016/j.ab.2007.10.027.

5. Källberg, M., Margaryan, G., Wang, S., Ma, J. & Xu, J. Raptorx server: A resource for template-based protein structure modeling. *Methods Mol. Biol.* (2014) doi:10.1007/978-1-4939-0366-5_2.
