# Supplementary material for: Structure-based insights into evolution of rhodopsins
Source: Commun Biol. 2021 Jun 30;4:821. doi: 10.1038/s42003-021-02326-4 (PMC8245419; doi:10.1038/s42003-021-02326-4)
Supplement: Supplementary file 4 — Reporting Summary [file 42003_2021_2326_MOESM4_ESM.pdf]

nature research

Corresponding author(s): Valentin Gordeliy

Last updated by author(s): May 13, 2021

Reporting Summary

Nature Research wishes to improve the reproducibility of the work that we publish. This form provides structure for consistency and transparency in reporting. For further information on Nature Research policies, see our Editorial Policies and the Editorial Policy Checklist.

Statistics

For all statistical analyses, confirm that the following items are present in the figure legend, table legend, main text, or Methods section.

n/a	Confirmed	
☐	☒	The exact sample size (*n*) for each experimental group/condition, given as a discrete number and unit of measurement
☐	☒	A statement on whether measurements were taken from distinct samples or whether the same sample was measured repeatedly
☒	☐	The statistical test(s) used AND whether they are one- or two-sided<br>*Only common tests should be described solely by name; describe more complex techniques in the Methods section.*
☒	☐	A description of all covariates tested
☒	☐	A description of any assumptions or corrections, such as tests of normality and adjustment for multiple comparisons
☒	☐	A full description of the statistical parameters including central tendency (e.g. means) or other basic estimates (e.g. regression coefficient) AND variation (e.g. standard deviation) or associated estimates of uncertainty (e.g. confidence intervals)
☒	☐	For null hypothesis testing, the test statistic (e.g. *F*, *t*, *r*) with confidence intervals, effect sizes, degrees of freedom and *P* value noted<br>*Give P values as exact values whenever suitable.*
☒	☐	For Bayesian analysis, information on the choice of priors and Markov chain Monte Carlo settings
☒	☐	For hierarchical and complex designs, identification of the appropriate level for tests and full reporting of outcomes
☒	☐	Estimates of effect sizes (e.g. Cohen's *d*, Pearson's *r*), indicating how they were calculated

Our web collection on statistics for biologists contains articles on many of the points above.

Software and code

Policy information about availability of computer code

Data collection	X-ray data collection: MXCube; UV-spectroscopy measurements: UVProbe 2.33; Ion translocation: Logger Lite 1.7.
Data analysis	Crystallography: XDS5, AIMLESS, REFMAC553, PHENIX, Coot, MoRDa, PyMOL, HOLLOW, PPM server. Time-resolved absorption spectra: MEXFIT, Origin.

For manuscripts utilizing custom algorithms or software that are central to the research but not yet described in published literature, software must be made available to editors and reviewers. We strongly encourage code deposition in a community repository (e.g. GitHub). See the Nature Research guidelines for submitting code & software for further information.

Data

Policy information about availability of data

All manuscripts must include a data availability statement. This statement should provide the following information, where applicable:

- Accession codes, unique identifiers, or web links for publicly available datasets
- A list of figures that have associated raw data
- A description of any restrictions on data availability

The protein coordinate and atomic structure factor have been deposited in the Protein Data Bank (PDB) under accession number 7BMH. All other data are available from corresponding author upon reasonable request.

Field-specific reporting

Please select the one below that is the best fit for your research. If you are not sure, read the appropriate sections before making your selection.

- [x] Life sciences
- [ ] Behavioural & social sciences
- [ ] Ecological, evolutionary & environmental sciences

For a reference copy of the document with all sections, see nature.com/documents/nr-reporting-summary-flat.pdf

Life sciences study design

All studies must disclose on these points even when the disclosure is negative.

Sample size	Sample sizes were determined based on prior literature and practices in the field. We did not use statistical methods a predetermine sample size.
Data exclusions	No data were excluded.
Replication	All attempts at replication were successful.
Randomization	The samples were not allocated into different groups, therefore the randomization was not required.
Blinding	Animal experiments were not performed in this study, so Investigators were not blinded to the experiment

Reporting for specific materials, systems and methods

We require information from authors about some types of materials, experimental systems and methods used in many studies. Here, indicate whether each material, system or method listed is relevant to your study. If you are not sure if a list item applies to your research, read the appropriate section before selecting a response.

Materials & experimental systems

n/a	Involved in the study	
[ ]	[x]	Antibodies
[ ]	[x]	Eukaryotic cell lines
[x]	[ ]	Palaeontology and archaeology
[x]	[ ]	Animals and other organisms
[x]	[ ]	Human research participants
[x]	[ ]	Clinical data
[x]	[ ]	Dual use research of concern

Methods

n/a	Involved in the study	
[x]	[ ]	ChIP-seq
[x]	[ ]	Flow cytometry
[x]	[ ]	MRI-based neuroimaging

Antibodies

Antibodies used	Penta·His Antibody, Qiagen, 34660, monoclonal;<br>Anti-Mouse IgG (Fc specific) - Alkaline Phosphatase antibody, Merck, A1418, polyclonal
Validation	The primary antibodies for immunostaining were validated by the manufacturer as descibed in the product datasheet

Eukaryotic cell lines

Policy information about cell lines

Cell line source(s)	Leishmania tarentolae host T7-TR cells were purchased from Jena Bioscience
Authentication	Cells have been authenticated by the vendors
Mycoplasma contamination	All cell lines tested negative for mycoplasma
Commonly misidentified lines<br>(See ICLAC register)	Cells are not listed in the database
